# TraPS-VarI: Identifying genetic variants altering phosphotyrosine based signalling motifs

**DOI:** 10.1038/s41598-020-65146-2

**Published:** 2020-05-21

**Authors:** Vijay Kumar Ulaganathan

**Affiliations:** 1 Department of Molecular Biology, Max Planck Institute of Biochemistry, Am Klopferspitz 18, Martinsried, 82152 Germany; 2 Present Address: Department of Neuroimmunology, Universitätsmedizin Göttingen, Von-Siebold-Str. 3A, Göttingen, 37075 Germany

**Keywords:** Immunosurveillance, Data mining, Cancer microenvironment, Multiple sclerosis, Molecular medicine

## Abstract

Patient stratification and individualized therapeutic strategies rely on the established knowledge of genotype-specific molecular and cellular alterations of biological and therapeutic significance. Whilst almost all approved drugs have been developed based on the Reference Sequence protein database (RefSeq), the latest genome sequencing studies establish the substantial prevalence of non-synonymous genetic mutations in the general population, including stop-insertion and frame shift mutations within the coding regions of membrane proteins. While the availability of individual genotypes are becoming increasingly common, the biological and clinical interpretations of mutations among individual genomes is largely lagging behind. Lately, transmembrane proteins of haematopoietic (myeloid and lymphoid) derived immune cells have attracted much attention as important targets for cancer immunotherapies. As such, the signalling properties of haematological transmembrane receptors rely on the membrane-proximal phosphotyrosine based sequence motifs (TBSMs) such as ITAM (immunoreceptor tyrosine-based activation motif), ITIM (immunoreceptor tyrosine-based inhibition motif) and signal transducer and activator of transcription 3 (STAT3)-recruiting YxxQ motifs. However, mutations that alter the coding regions of transmembrane proteins, resulting in either insertion or deletion of crucial signal modulating TBSMs, remains unknown. To conveniently identify individual cell line-specific or patient-specific membrane protein altering mutations, we present the Transmembrane Protein Sequence Variant Identifier (TraPS-VarI). TraPS-VarI is an annotation tool for accurate mapping of the effect of an individual’s mutation in the transmembrane protein sequence, and to identify the prevalence of TBSMs. TraPS-VarI is a biologist and clinician-friendly algorithm with a web interface and an associated database browser (https://traps-vari.readthedocs.io/).

## Introduction

The membrane protein family is one of the highly coveted protein targets for therapeutic interventions. It is becoming increasingly evident that areas of the genome that differ between individuals (mutations) play a substantial role in disease susceptibility, disease progression and can determine therapeutic outcomes. Genetic mutations varying the coding sequence of integral membrane proteins are potent mediators of physiological variations that can result in life threatening diseases^1–4^. Due to the nature of sequence variations in the extracellular and intracellular segments of the membrane proteins, a wide spectrum of biologically and clinically relevant protein interactions, with varied affinities to natural and synthetic molecules, are anticipated^5,6^. Mutations in the coding regions of integral membrane proteins in humans may alter not just the therapeutic efficacy but also determine the safety of drugs^7^. The knowledge of inter-individual genetic differences within the coding regions of transmembrane proteins holds great promise in the emerging field of individualized medicine.

Surface molecules of immune cells harbour membrane-proximal TBSMs in the cytosolic domains, to either augment, dampen or modulate downstream signalling by serving as docking sites for cytoplasmic kinases, phosphatases and transcription factors, thereby triggering and coordinating signal transducing events^8,9^. Membrane-proximal TBSMs, namely ITAM with the consensus sequence – [YxxI/Lx_(6–12)_YxxI/L], ITIM with the consensus sequence – [S/I/V/LxYxxI/V/L] and STAT3-recruiting motif with the consensus sequence - [YxxQ] motif, all trigger evolutionarily conserved proximal signalling events that feed an extensive network of phosphorylation cascades in the cytoplasm. While phosphorylated ITAMs serve as docking sites for tandem Src Homology 2 (SH2) domains of Syk family kinases^10^ phosphorylated ITIMs recruit tyrosine phosphatases to inner membranes^11^ and the phosphorylated YxxQ motifs serve as docking sites for SH2 domains of STAT3 transcription factors^12^. In mammals, ITAMs, ITIMs and membrane-proximal YxxQs, are found in a large number of cell surface proteins involved in the regulation of the immune system, bone and brain homeostasis^13–15^. Lately, TBSMs are also reported to play a significant role in cancer development^16^ and as potential modifiers of cell-based therapies^17–19^. The qualitative and quantitative roles of tyrosine-based signalling motifs in membrane proteins of the immune system is well characterized^20^. Over recent years, knowledge on the prevalence of the mutations altering transmembrane protein sequences have also enormously expanded^21,22^. However, the prevalence of mutations that alter the occurrence of membrane-proximal TBSMs in the human genome is unknown. This information may provide valuable mechanistic insight into the incidence and the clinical progression of complex diseases such as autoimmunity and various cancers.

To determine the spectrum of mutations prevalent in cell surface proteins and to facilitate identification of rare mutations altering proximal signalling phosphotyrosine motifs, we developed TraPS-VarI. TraPS-VarI is a simple annotation tool for conveniently identifying mutations that affect membrane protein sequences by using human genotyping data in the variant call format (vcf) file. An update is planned in the future to use whole human genome data in binary alignment map (bam) format as inputs. The underlying python script for TraPS-VarI (available via gitlab: https://gitlab.com/VJ-Ulaganathan/TraPS-VarI) is easy to install and runs locally.

## Results

### Mapping of genotype data to membrane proteins

TraPS-VarI is the first tool of its kind to map allelic variants (recorded in vcf files) to the sequence of transmembrane proteins (Supplementary Fig. S1), and more specifically, to the position within the domain of the proteins. It can also predict the effect of the mutation on the membrane-proximal TBSMs (Supplementary Fig. S2).

TraPS-VarI traces mutations through to their effects on the coding regions of membrane proteins, by processing the vcf file, line by line, using a mapping path that runs through nodes which include the Genome Reference Consortium (GRC), Human Genome build versions h37 and h38. It takes the position and matches this against coding regions in the RefSeq database^23^. It then matches the coding DNA sequence (CDS) to its appropriate Universal Protein knowledgebase (UniProt)^24^ entry, modifies the CDS according to the mutation, and re-translates the resulting CDS into the mutated full length protein sequence. The effect of the mutation is derived from the difference between those two entries. In its current version it only matches against UniProt’s main entries and not their isoforms (support for this is planned). It also cross checks the position and mutation in the database of single nucleotide polymorphisms (dbSNP)^25^ and if the mutation is present, it adds the dbSNP reference SNP cluster (rs) identifier (id) to the entry. TraPS-VarI maps all genetic alterations recorded in the vcf file including normal SNPs, frameshift, stopinsertion and start-deletion mutations across the full length protein sequences, and the tool can be filtered to show specific families of proteins, in the case of this example, only transmembrane proteins. For all transmembrane proteins mapped, associated therapeutic and pharmacologic agents available in the DrugBank database^26^ and information about entries under clinical investigation, available in the Therapeutic Target Database & Clinical Trials^27^ are extracted.

### Prevalence of deleterious mutations in cell surface proteins

**Figure 1:**
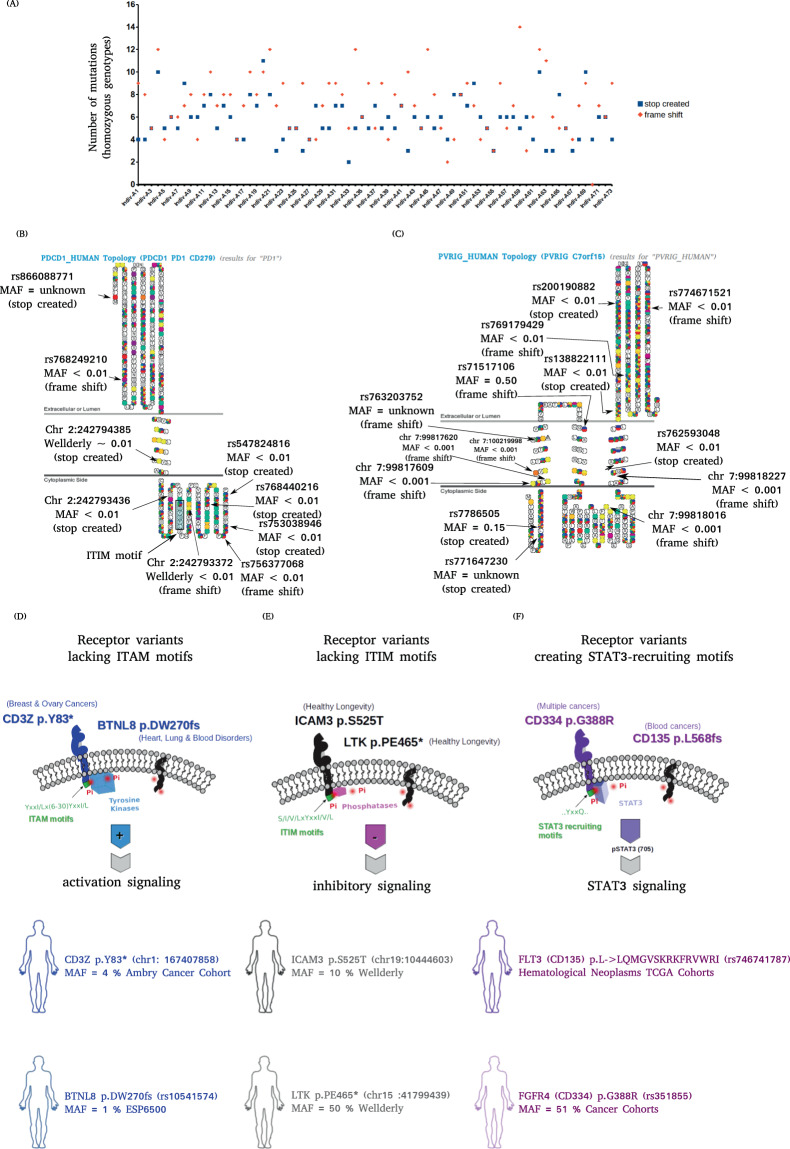
Prevalence of deleterious mutations in cell surface proteins. (**A**) Prevalence of stop created and frame shift genetic variations encoding human membrane proteins in homozygous genotypes among individual genomes (n = 73). (**B**) Graphical display of the mapping of frame shift and stop creating genetic variation on the topology diagram of CD8 T cell co-inhibitory receptors namely PDCD1 and PVRIG. When a variation was identified by multiple studies a multi-coloured circles are depicted where yellow denotes results from Wellderly studies. (**D–F**) Prevalence of individual-specific immunoreceptor variants among the diseased and healthy population groups. Germline alleles encoding a receptor variant that impact the presence of membrane-proximal tyrosine sequence motifs such ITAM, ITIM and STAT3-docking motifs are depicted along with the minor allele frequencies.

The human genome encodes an estimated 5195 proteins with at least one transmembrane domain, including type I, type II and multi-pass classes of membrane protein (Supplementary Fig. S3). Almost every region in the full length protein sequence of the membrane proteins are susceptible to single nucleotide polymorphic (SNP) mutations. Consequently, a wide spectrum of structural variations are proximal to the cell membrane and at the molecular level, interaction variations are anticipated. Interestingly, signal-peptide and transmembrane segments exhibit the highest resistance to genetic alterations, suggesting that mutations here may be lethal, or that plausibly cannot be compensated by any complementary counter mutations (Supplementary Figs. S4–S13) (https://vj-ulaganathan.github.io/Supplementary Fig.S4-S13.pdf). Given the prevalence of frame-shift and stop-insertion mutations in all other domains of membrane proteins including extracellular domains of cell surface molecules, we anticipate that this should be an important point of concern for biologists working with human cells and clinicians prescribing target-specific therapeutic agents such as monoclonal antibodies or recombinant cytokines (Supplementary Fig. S14, Table S1). Furthermore, looking into the genotypes of 73 random individuals, it is evident that on average an individual genome harbours about 5 to 7 deleterious mutations (7 frameshift & 5 stop insertion) in homozygous genotypes (Fig. 1A**)**. Intriguingly, such truncated variants are prevalent among olfactory receptors, gustatory receptors, ion channels and cell surface molecules mediating immune cell-cell interactions (Supplementary Table S2, Fig. S2). Furthermore, a comprehensive analysis of open source human genotyping datasets revealed high prevalence of truncation variants affecting T cell receptor signalling in the cancer and wellderly cohorts (MAF > 1%) (Fig. 1B–F). A comparative analysis focussed on the distribution of mutations in specific membrane protein domains, from Ensembl, 1000 genome, dbSNP, Exome Aggregation Consortium (ExAC), deCODE, AmbryShare, Wellderly and personal genome project (PGP) datasets indicated variations in all domains of the human transmembrane proteins. Interestingly, the analysis revealed a relatively higher percentage of mutations (~4% of total mutations) affecting the N-terminus ‘signal sequence’ in the genomes of the only longevity dataset of the Wellderly study cohort, while the percentage in all other datasets analysed tend to remain less than 2% (Supplementary Fig. S15A,B). The relevance of this high prevalence of mutations in the signal sequence region in this longevity cohort is puzzling.

### Identification of mutations altering phosphotyrosine signalling motifs

A comprehensive TraPS-VarI analysis of the available human genotyping datasets traced the insertion and deletion of consensus tyrosine-based signalling motifs in the membrane-proximal domains of bitopic membrane proteins. Although, the frequency of mutations affecting the membrane-proximal domains is low in the general population, their identification resulted in a substantially expanded list of potential immunoreceptor variants (Supplementary Table S3). To name a few, chr17:72691356 - G/A encoding CMRF35-like molecule 1 (CLM1) p.S251Y creates ITAM at position 251 (251, ‘YLTLGAEDQEPTYCNMGHLSSHLPGRGPEEPTEYSTIS’), and chr5:38932983 - C/T encoding Oncostatin-M-specific receptor (OSMR) p.H793Y creates ITAM at position 771(771, ‘YPDIPDPYKSSILSLIKFKENPYLIIMNVSDCIPDAIE’). Whether such rare receptor variants viz., CLM1 p.S251Y and OSMR p.H793Y harbouring ITAMs have functional and clinical relevance in modifying immune responses is unknown and worth studying further. Similarly, genetic alterations in the molecules of the immune and nervous systems that result in receptor variants harbouring ITIM motifs are listed in Supplemental Table 3. In the genomes of cancer cohorts, genetic alterations are also found to either destroy the existing TBSMs or leave the TBSM intact while truncating a major portion of the cytoplasmic domains. For instance, chr 1:167407858 - C/T encoding the CD3Z p.Y83* variant deletes ITAM motifs (cancer cohort, MAF ~ 4%), chr 15:41799439 - AGG/A encoding the Leukocyte tyrosine kinase receptor (LTK) p.PE465* variant deletes ITIM motifs (Wellderly cohort, MAF ~ 50%) whereas rs746741787 - CAG/TCCAG encoding the fms-like tyrosine kinase 3 (FLT3) p.I638* truncation variant leaves intact the two STAT3 recruiting TBSMs namely namely ‘566-YKKQ’ and ‘572-YESQ’ while deleting the cytoplasmic domain (Fig. 1D–F).

**Figure 2:**
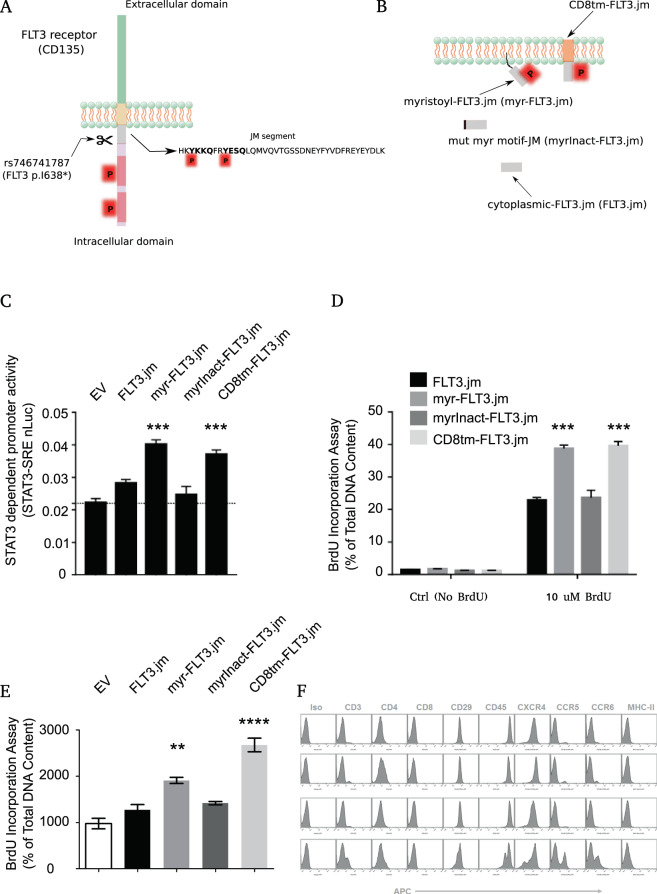
Identification of mutations altering phosphotyrosine signalling motifs. (**A**) Graphical depiction of a phosphotyrosine-altering genetic variant in FLT3 juxtamembrane segment (FLT3.jm). Rs746741787 is a indel variation truncating the cytoplasmic segment of FLT3. Phosphotyrosines are highlighted in red. (**B**) Graphical summary illustrating synthetic biology strategies to express TraPS-VarI predicted STAT3-recruiting motifs in FLT3.jm as either membrane-proximal or cytoplasmic molecules to answer if computationally predicted motifs are biologically relevant. (**C**) STAT3 dependent promoter activity in 3T3NIH cell lines expressing indicated constructs measured by Nano-glo dual luciferase assay using pSRE-nano luciferase and pTK-firefly luciferase as reporter constructs. Shown are representative of three independent experiments each performed in 5 measurement replicates. Value P  <  0.0001 is denoted by *** respectively. (**D,E**) Assessment of cell proliferation by the BrdU incorporation assay. Serum-starved 3T3NIH (**D**) and BW5147 (**E**) cells were incubated overnight with 10 μM BrdU and the DNA incorporation was measured using anti-BrdU mAb by flow cytometry. Shown are representative of three independent experiments each performed in 5 measurement replicates.Values of P  <  0.001, P  <  0.0001 and P < 0.00001 are denoted by **, *** and **** respectively. (**F**) Expression profiling of indicated surface markers by flow cytometry analyses in BW5147 cell lines.

To experimentally validate whether ‘566-YKKQ’ and ‘572-YESQ’ motifs in the FLT3 p.I638* variant (Fig. 2A) predicted by TraPS-VarI are biologically relevant, the short peptide sequence matching the FLT3 membrane-proximal segment from 564–602 amino acids (FLT3.jm) was investigated by exogenous expression in human (HEK293T) and mouse cell lines including those of non-hematopoietic (3T3NIH) and hematopoietic (BW5147) origin. FLT3.jm was transiently expressed in HEK293T cells and as expected, showed different localisation depending on the tag. myr-FLT3.jm expression produced a membrane-anchored molecule via an N-terminal myristoylation sequence; CD8tm-FLT3.jm expression produced a membrane-embedded molecule via N-terminal fusion to CD8 transmembrane helix; myrInact-FLT3.jm expression produced a peptide with a mutated myrisotylation sequence and thus the expressed peptide had no specific localisation to membranes, and as control to show that FLT3 had no localisation alone, FLT3.jm expression produced a peptide which had no specific localisation to membranes (Fig. 2B). For membrane-proximal recruitment of STAT3 by tyrosine-based sequence motifs, the tyrosines in the ‘YxxQ’ motifs must be phosphorylated. To determine if ‘566-YKKQ’ & ‘572-YESQ’ motifs were phosphorylated, phosphotyrosine levels were assessed by flow cytometry analysis of HEK293T cells transfected with FLT3.jm, myr-FLT3.jm, myrInact-FLT3.jm and CD8tm-FLT3.jm expression constructs. A significant increase in phosphotyrosine levels was detected in cells expressing ‘566-YKKQ’ & ‘572-YESQ’ motifs in either membrane-anchored myr-FLT3.jm or membrane-embedded CD8tm-FLT3.jm molecules indicating the potential to function as STAT3-docking sites proximal to the inner leaflet of cell plasma membranes (Supplementary Fig. S16A–D). Furthermore, expression of myr-FLT3.jm or CD8tm-FLT3.jm resulted in significant upregulation of STAT3-dependent promoter activity (Fig. 2C) and increased cell proliferation as measured by BrdU incorporation (Fig. 2D) in 3T3NIH cells, suggesting enhancement of the STAT3 signalling pathway. Similarly, in BW5147 cell lines, expression of FLT3.jm as either membrane-anchored or membrane embedded molecules not only enhanced the proliferation (Fig. 2E) but also altered the cell surface expression of chemokine receptors (Fig. 2F**)**. Interestingly, when FLT3.jm was expressed as a cytoplasmic molecule, lacking specific localisation to inner cell membranes, it lacked any biological activity, causing no notable alterations in STAT3 signalling, highlighting that only membrane-proximal “YxxQ” motifs are biologically relevant. These experimental results suggest that membrane-proximal ‘YxxQ’ motifs that were left intact in the FLT3 p.I638* variant by the frame shift mutation rs746741787 are biologically relevant and that FLT3 p.I638* is a gain-of-function receptor variant. This is the first report describing the gain of biological activity by the membrane-proximal STAT3-recruiting YxxQ phosphotyrosine motifs in the FLT3 p. I638* variant, which conversely has been predicted to be a loss-of-function variant by Sorting Tolerant From Intolerant (SIFT)^28^ and Polymorphism Phenotyping v2 (PolyPhen-2)^29^ gene variant annotation algorithms. This will be commented on the in the Discussion and Conclusion.

### Usage of TraPS-VarI and its associated database browser

To circumvent the need for knowledge of installation and execution of TraPS-VarI on the command line, we present the computational tool as a web application as well (accessible from https://traps-vari.org/#section-2). It is based on the Django frame-work and runs on the public server, thus making it platform agnostic and conveniently accessible. Extensive documentation with illustrations is included in the linked pages. In contrast to the locally executable python module which generates static text files, the Django framework based web application is interactive and allows the user to select a defined motif, sort the columns and re-analyse the resulting data for allele distribution or clinical studies. The application prefers compressed or archived forms of vcf input files. The tabulated results can be conveniently queried over the geography of the genetic variants browser to view the distribution of alleles across the global populations. Up-to-date information on ongoing clinical trials can also be accessed using the row-wise hyperlinked URLs produced on the results table (Supplementary Fig. S17). Furthermore, the associated TraPS-VarI Database Browser (accessible from https://www.traps-vari.org/trapsDbBrowser/) displays the spectrum of alterations by overlaying amino acid changes on topology diagrams using the aggregated genotyping datasets namely: 1000 genomes, dbSNP, Ensembl, Exome Aggregate Consortium, deCode, Harvard Personal Genome Project, Cancer Cell Line Encyclopedia, Catalogue Of Somatic Mutations in Cancer, The Cancer Genome Atlas, National Cancer Institute (NCI)-60 cell lines, Ambryshare and Wellderly.

The results of comprehensive receptor-based human genetic variation data analyses is thus made publicly accessible under the TraPS-VarI database browser (TraPS-VarI Db). Browsing receptor-specific data using the TraPS-VarI Db graphical display facilitates easy identification of biologically and clinically relevant cell surface variants that otherwise may be a cumbersome task. For instance, a quick search for chr1:1147125 displays the creation of the STAT3 docking site in TNFRSF4 p. R241Q receptor variant; for 9:35650538 the deletion of the ITIM motif in SIT1 p.W66* receptor variant is displayed; for chr13:111995143 creation of a new ITAM motif in TEX29 p.D94Y receptor variant is displayed; for chr12:9885707 truncation of entire extracellular and membrane targeting transmembrane segment in CLECL1 p. S52fs receptor variant is displayed, and for chr2:203867991 a point mutation in the signal sequence of CTLA4 p.T15A receptor variant is displayed (Supplementary Fig. S18).

## Discussion and Conclusion

Understanding genetic variation in drug targets is of vital significance for tailoring drug prescriptions based on individual genotypes, to maximize efficacy and safety while reducing side effects. The present understanding of receptor biology in humans comes in large from studying protein sequences of animal models or sequences cloned from certain human derived cell lines. In most cases, investigators rely on sequences provided by the RefSeq database, regularly being updated with new genetic variation data^30^. A data explosion driven by the renaissance of computational advancements call for a human genotype-centric approach not just to incorporate new information but also to address fundamental questions in the context of human biology that are difficult to discern from animal model studies alone^31^. Several recent studies have shown that protein coding genetic variations, including structural variations and loss-of-function alterations, are widely dispersed throughout the human genome^32–35^. Recently, using the latest developments in exome sequencing technologies, a comprehensive catalogue of mutations across multiple human populations have been generated, called the Genome Aggregation Database (gnomAD)^36–40^. An important message drawn from this large-scale aggregation of more than 140,000 human exomes was the widespread prevalence of deleterious mutations in the human population^41^. It is now recognized that gnomAD data has broad utility not just for population genetics, disease association and diagnostic screening but also for facilitating gene variant discovery and clinical interpretations of gene variants in clinical trials.

In this work, we describe TraPS-VarI, a biologist and clinician friendly annotation tool for identifying membrane protein variants using the individual cell line-specific or patient-specific genotyping datasets, which has become commonplace in many clinical labs. Studying the allelic variants of membrane proteins can provide valuable insights into the genotype-based aspects of signalling biology as well as inter-individual variation in the therapeutic outcomes.

TraPS-VarI allows convenient identification of membrane protein altering mutations by using individual or aggregated genotyping datasets as vcf files. In general, mutations are found dispersed throughout the coding regions of all membrane proteins of the human genome. By examining various open source human genotyping datasets, we curated all deleterious mutations resulting in truncation of membrane proteins, which are potentially capable of modifying therapeutic outcomes to almost all of the Food and Drug Administration (FDA)-approved therapeutic monoclonal antibodies available in the TraPS-VarI Db browser.

Among the deleterious alleles in the general population, the most common alleles were found to be truncating membrane proteins of olfaction, gustation and immune responses. Among the truncation variants affecting T cell receptor signalling, deletion of ITAM motifs and creation of STAT3-recruiting YxxQ motifs was commonly found in the cancer cohorts while deletion of ITIM motifs was commonly found in the wellderly cohorts. The question as to why these mutations are prevalent in different subsets of population, is intriguing; whether compensatory roles among chemosensory and immune signals play a role in disease resistance mechanisms to common human diseases or mate selection in human population remains unknown^42^.

Another interesting finding that emerged from this work was the high degree of variation found in the N-terminus signal-sequence domains of membrane proteins among the elderly longevity cohorts. Cleavable N-terminal signal peptides of membrane proteins play a key role in targeting and integration of proteins to their target membrane, for example targeting newly synthesized receptors into the Endoplasmic Reticulum^43^. Genetic alterations in signal peptide sequence are pleiotropic in nature and it has been suggested that some may affect the homeostasis of the endoplasmic reticulum with ageing^44^, although it is plausible that such mutations are well-tolerated in the genomes of longevity cohorts, but this remains to be clarified.

Cancer therapy has been revolutionized by immunotherapies that target immune cells instead of tumour cells, while being highly effective against many cancer types^45^. This has sparked wide-spread interest in the scientific community. However, to date only a subset of patients are benefiting from cancer immunotherapy^46^. Subsequently, there is enormous interest in identifying biomarkers that can help to identify the right patients who will respond better to immunotherapies^47^. Among the factors that may determine this, genetically determined variation in the patient immune system is often neglected^48^, even though genetic diversity causes 20–40% of the variation between the immune systems of individuals^49–51^. In this regard, TraPS-VarI is timely as it facilitates identification of genetic variations that impact the proximal signalling in immune cells via the phosphotyrosine motifs in adapter molecules, immunoreceptors and transmembrane molecules. Whether such genetic alterations can serve as a marker for predicting the prognosis of immunotherapies is an open question that interests us as well. In this study, it was noted that ITAMs and ITIMs occur in various transmembrane proteins previously not recognized as *bonafide* immunoreceptors. Additionally, it is revealed that the rare rs746741787–TCCAG allele is a gain-of-function mutation that enhances STAT3 signalling via the intact membrane proximal YxxQ motif in the FLT3 p.I638* receptor variant. In contrast, many public annotation algorithms assign rs746741787 as a loss-of-function variant. Currently used genetic variation annotating algorithms do not take into account the biological relevance of genetic mutations impacting consensus proximal signalling motifs. It is foreseeable that future studies investigating genetic variations that alter proximal signalling in immune cells will uncover potential new targets for immunotherapeutic intervention and help to design innovative signalling molecules for cell-based therapies.

In order to disentangle the role that human membrane protein variants contribute to inter-individual variations in physiology, disease and therapeutic outcomes, the huge gap between data generation and biological interpretation has to be narrowed. It is necessary to learn with certain levels of accuracy the effects of each variant at the molecular and cellular levels before extrapolating to the level of the organism. In this regard, the vision of precision medicine cannot be realized to its full extent without practising precision biology at the bench-side. The receptor variants discussed here underscore the importance of taking into consideration individual genome-specific alterations while formulating experimental hypotheses for realizing the goal of personalized medicine. To this end the tool and the resource presented here is valuable and poised to further a vision for precision biology framework.

## Methods

### Cell lines

3T3NIH (LGC/ATCC, #CRL-1658), GP + E-86 packaging cell line (LGC/ATCC, #CRL-9642) and AKR/J mouse thymoma BW5147 (ATCC, #TIB47) cell line was obtained from ATCC was authenticated and confirmed to be free-from mycoplasma. Cells were cultivated in Dulbecco’s Modified Eagle’s Complete Medium with 10% FCS.

### Plasmids

Sleeping beauty based expression constructs namely pCMV-SB100X and pCAG-FLT3JM40-P2A-Clover-T2A-Puro, pCAG-mCD8tm-FLT3JM40-P2A-Clover-T2A-Puro, pCAG-myrFLT3JM40-Clover-T2A-Puro and pCAG-myrMUT-FLT3JM40-P2A-Clover-T2A-Puro, encoding FLT3 juxtamembrane phospho-tyrosine motifs driven by cytomegalovirus and chicken beta actin promoter respectively, were generated for working with easy to transfect 3T3NIH cells. The MSCV (Murine Stem Cell Virus) retroviral plasmid, pMSCVneo (Clontech, #631461) was generated for working with difficult to transfect BW5147 thymoma cell lines. To construct retroviral expression plasmids, namely pMSCVneo-FLT3JM40-P2A-Clover-T2A-Puro, pMSCVneo-mCD8tm-FLT3JM40-P2A-Clover-T2A-Puro, pMSCVneo-myrFLT3JM40-Clover-T2A-Puro and pMSCVneo-myrMUT-FLT3JM40-P2A-Clover-T2A-Puro, polycistronic inserts were generated using gene synthesis services (Eurofins Genomics) and cloned between EcoRI and BgII sites in pMSCVneo vector.

Expression plasmids are made available for distribution from Addgene.

### Retroviral transduction of thymoma cells

To stably express phosphotyrosine motifs in BW5147 (ATCC, #TIB47) cell lines, GP + E-86 packaging cells (LGC/ATCC, #CRL-9642) were used. BW5147 were transduced by co-cultivation with adherent GP + E-86 retrovrial packaging cell lines as previously described. GP + E-86 cells were transfected using Lipofectamine 2000 (Life Science Technologies, #11668027) with retroviral plasmids and selected with puromycin for a week. Clover-positive packaging cell lines were sorted by flow cytometry prior to co-cultivation.

### Flow cytometry

To determine expression of surface markers, single cell suspensions in PBS containing 0.1 mM EDTA and 2% FCS were stained with the following directly conjugated antibodies: APC anti-mouse CD3 (BioLegend, Cat# 100312, Clone-134-2C11), APC Armenian Hamster IgG Isotype Ctrl Antibody (BioLegend, Cat# 400911, Clone-HTK888), APC anti-mouse CD4 Antibody (BioLegend, Cat# 100516, Clone-L3T4, T4), APC Rat IgG2a, κ Isotype Ctrl Antibody (BioLegend, Cat# 400511, Clone-RTK2758), APC anti-mouse CD8a Antibody (BioLegend, Cat# 100712, Clone-53-6.7), APC anti-mouse/rat CD29 Antibody (BioLegend, Cat# 102215, Clone-HMβ1-1), APC anti-mouse CD184 (CXCR4) Antibody (BioLegend, Cat# 146507, Clone-L276512), APC anti-mouse CD195 (CCR5) Antibody (BioLegend, Cat# 107011, Clone-HM-CCR5), APC anti-mouse CD196 (CCR6) Antibody (BioLegend, Cat# 129813, Clone-29-2L17), APC anti-mouse I-Ab Antibody (BioLegend, Cat# 116417, Clone-AF6-120.1), APC Mouse IgG2a, κ Isotype Ctrl Antibody (BioLegend, Cat# 400221, Clone-MOPC-173), APC anti-mouse CD45 Antibody (eBioscience, Cat# 17-0451-82, Clone-30-F11), APC Armenian Hamster IgG Isotype Control (eBioscience, Cat# 17-4888-82, Clone-eBio299Arm) and APC CD90.1 (Thy-1.1) Monoclonal Antibody (eBioscience, Cat# 17-0900-82, Clone-HIS51).

For intracellular staining, cells were fixed in 4% PFA for 15 mins at room temperature. After one time wash in PBS, cells were resuspended in cell permeabilization buffer (0.3% Triton X-100 in PBS containing 1 mM EDTA & 2% FCS) and incubated at room temperature for 30 mins. Hereafter, cells were washed and incubated in cell permeabilization buffer for subsequent primary antibody staining with phospho-Tyrosine mouse mAb (Cell Signaling Technology, Cat# 9416, Clone-P-Tyr-102) and HA-Tag Rabbit mAb (Cell Signaling Technology, Cat# 3724, Clone-C29F4).

### BrdU proliferation assay

For flow-cytometry-based detection of BrdU incorporation in 3T3NIH, serum-starved cell lines were incubated in RPMI culture medium containing 1× BrdU. After indicated time points, cells were detached and washed once in PBS. Cells were fixed and permeabilized with 70% ice cold ethanol for 5 mins at room temperature and rinsed two times in PBS followed by treatment with 1.5 M HCl at room temperature for 30 mins. Washed cells were stained for BrdU using Mouse anti-BrdU antibody (Cell Signaling, #5292) or mouse isotype control and for DNA using propidium iodide 10 μg/mL. Allophycocyanin-conjugated anti-mouse (Dianova, #115-136-146) was used as secondary antibody and measured in a flow cytometer.

### STAT3-dependent promoter activity assay

3T3NIH cells (cultured at 37 °C, 7% CO2 in RPMI containing 4.5 g l − 1 glutamine and 10% FBS) were transiently transfected with reporter plasmids Cignal STAT3 Reporter (luc) kit (Qiagen, #CCS9028L), pTk-Renilla and plasmids pCAG-FLT3JM40-P2A-Clover-T2A-Puro, pCAG-mCD8tm-FLT3JM40-P2A-Clover-T2A-Puro, pCAG-myrFLT3JM40-Clover-T2A-Puro and pCAG-myrMUT-FLT3JM40-P2A-Clover-T2A-Puro using Lipofectamine 2000 (Life Science Technologies, #11668027). Luciferase was measured with the Dual Glo Luciferase Assay System (Promega, #E1910) according to the manufacturer’s instructions. Briefly, Dual Glo Luciferase Reagent was added to the cells and, after incubation for 10 min, firefly luciferase activity was measured with a luminometer (EG&G Berthold Technologies, LB96v). Reactions were stopped by treatment for 10 min with Dual-Glo Stop and Glo Reagent and renilla luciferase activity was then measured. For some samples where increased sensitivity was required, a Nano-Glo Dual Luciferase Reporter Assay Prototype Kit (Promega, #N1110) was used.

### Data sources and data import

In all cases, data were obtained from primary sources and parsed with python to a tab-delimited file and then imported into a MySQL (for TraPS-VarI) and sqlite database (for TraPS-VarI Database Browser) with a python interpretor. The following databases were utilized; NCBI Reference Sequence (RefSeq)^23^, Universal Protein Resource (UniProt)^24^, 1000 Genomes project^52^, Single Nucleotide Polymorphism database (dbSNP)^25^, Ensembl Variation database^53^, NHLBI Exome Sequencing Project (ESP)^54^, International HapMap Project^55^, Exome Aggregation Consortium (ExAC)^36^, National Cancer Institute (NCI)−60 cell lines^56^, Cancer Cell Line Encyclopedia (CCLE)^57^, Catalogue Of Somatic Mutations in Cancer (COSMIC)^58^, The Cancer Genome Atlas (TCGA), AmbryShare (Ambry Genetics), the Scripps Translational Science Institute Wellderly genome sequencing project^59^, Harvard Personal Genome Project^60^, DrugBank^26^, Therapeutic Target Database (TTD)^27^ and ClinicalTrials repository. Drug entries matched to UniProt Entry were imported into the TraPS-VarI MySQL database.

### Comparative analysis of cancer versus wellderly cohorts

To demonstrate the utility of TraPS-VarI for identification of novel membrane protein variants data from AmbryShare and Wellderly cohorts were parsed into vcf file format for analyses by TraPS-VarI via the command line. Comparison of the results based on allele frequencies in respective cohorts revealed several interesting membrane protein variants that are potentially unique to cancer patients.

### Implementation

TraPS-VarI is built on python with MySQL as the primary data store. The web application is built on Django framework hosted with Nginx-Gunicorn web-server on a server running Ubuntu server16.04. The TraPS-VarI Database Browser web application aggregates genetic variation data and overlays the amino acid alteration information on the membrane protein topology diagram.

### Access

TraPS-VarI web interface allows membrane protein-centric analyses of human individual’s genotype dataset through simple one step submit button. The email is optional if long waiting times are not of concern. The status of the submitted analysis can be checked by pasting the JobID. The emailed results page indicates all genetic alleles for the individual-specific membrane proteins in the tabular format. In addition the sortable table displays tabs for DrugBank and Therapeutic Target Database entries for the matching UniProt entries. The ‘Search’ form allows prefiltering of results. The results can be downloaded as tab-separated file or copied into clipboard for pasting into suitable editor. The results can re-analysed for additional information such as allele frequencies and protein target specific clinical trials by clicking the ‘send to FreqInPop’ or ‘send to TargetOnTrials’ respectively. The genetic variations in membrane proteins aggregator TraPS-VarI database browser is a web interface for browsing the spectrum of protein sequence alterations displayed by overlaying on the topology diagram. The hyperlinked amino acids display database specific allele information including presence of membrane-proximal tyrosine motifs.

### Statistics

Statistical analyses were performed using Prism software (GraphPad Prism). Biological and measurement replicates are indicated in the corresponding figure legends and statistical methods. For two-group comparisons, unpaired t-test was used. All P values are two-tailed; the criterion for statistical significance was P  <  0.05. Values of P  <  0.05, P  <  0.001, P  <  0.0001 and P < 0.00001 are denoted by *, **, *** and **** respectively. All data are represented as means either ± s.d.

## Supplementary information

Supplementary Information.

Supplementary Table 1.

Supplementary Table 2.

Supplementary Table 3.

## Data Availability

TraPS-VarI is an open source initiative available in the GitLab repository: https://gitlab.com/VJ-Ulaganathan/TraPS-VarI, and the associated membrane protein variation repository is accessbile under the url: https://www.traps-vari.org/trapsDbBrowser/.
