# Supplementary material for: TraPS-VarI: Identifying genetic variants altering phosphotyrosine based signalling motifs
Source: Sci Rep. 2020 May 21;10:8453. doi: 10.1038/s41598-020-65146-2 (PMC7242328; doi:10.1038/s41598-020-65146-2)
Supplement: Supplementary file 1 — Supplementary Information. [file 41598_2020_65146_MOESM1_ESM.pdf]

Supplementary Information

TraPS-VarI: Identifying genetic variants altering phosphotyrosine based signalling motifs

Vijay Kumar Ulaganathan[1,2,3,*]
[1] Department of Molecular Biology, Max Planck Institute of Biochemistry,
Am Klopferspitz 18, Martinsried, 82152, Germany
Tel: ++49-89-85782511 & Email: *ulaganat@biochem.mpg.de*

[2] Department of Neuroimmunology, Universitätsmedizin Göttingen,
Von-Siebold-Str. 3A, Göttingen, 37075, Germany
Tel: ++49-551-39-61142 & Email: *vijaykumar.ulaganathan@med.uni-goettingen.de*

[3] Current address.
* To whom correspondence should be addressed.

Supplementary Table 1

Source data for the identification and cataloguing of deleterious mutations that may potentially alter the drug efficacy. List of currently available FDA-approved therapeutic monoclonal antibodies and the associated drug targets.

Supplementary Table 2

List of individual-specific frameshift and stop creating homozygous mutations indicating the prevalence of such deleterious germline mutations in the general population (n=74). The genotypic datasets were kindly provided by the Harvard Personal Genome Project (https://my.pgp-hms.org/).

Supplementary Table 3

List of newly identified immunoreceptors based on TraPS-VarI analysis of publicly available human variome datasets.

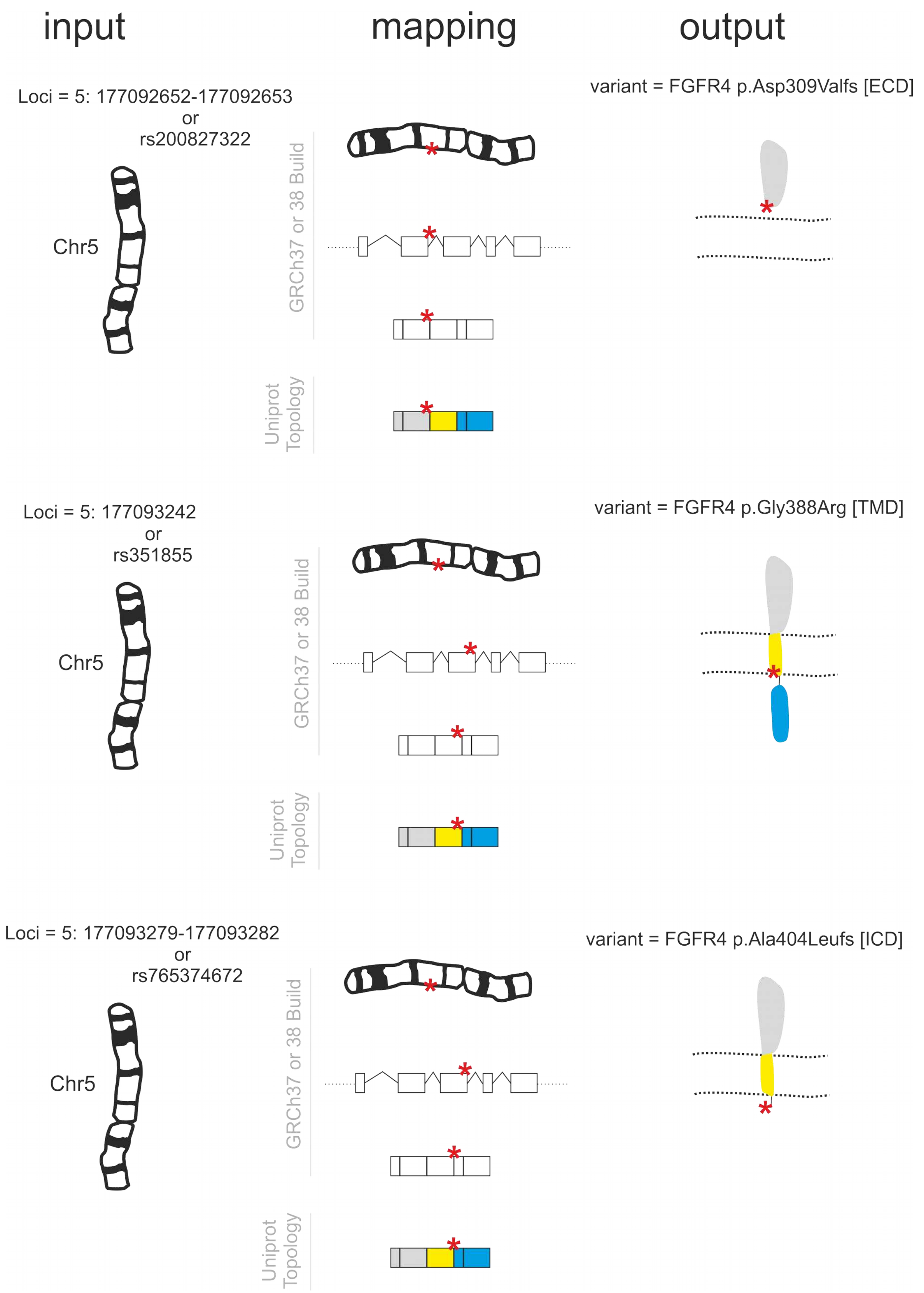

Supplementary Fig. S1 Mapping of the altered allele

Mapping of the altered allele recorded in the vcf file to membrane protein domain. For illustration, three variants of FGFR4 is depicted.

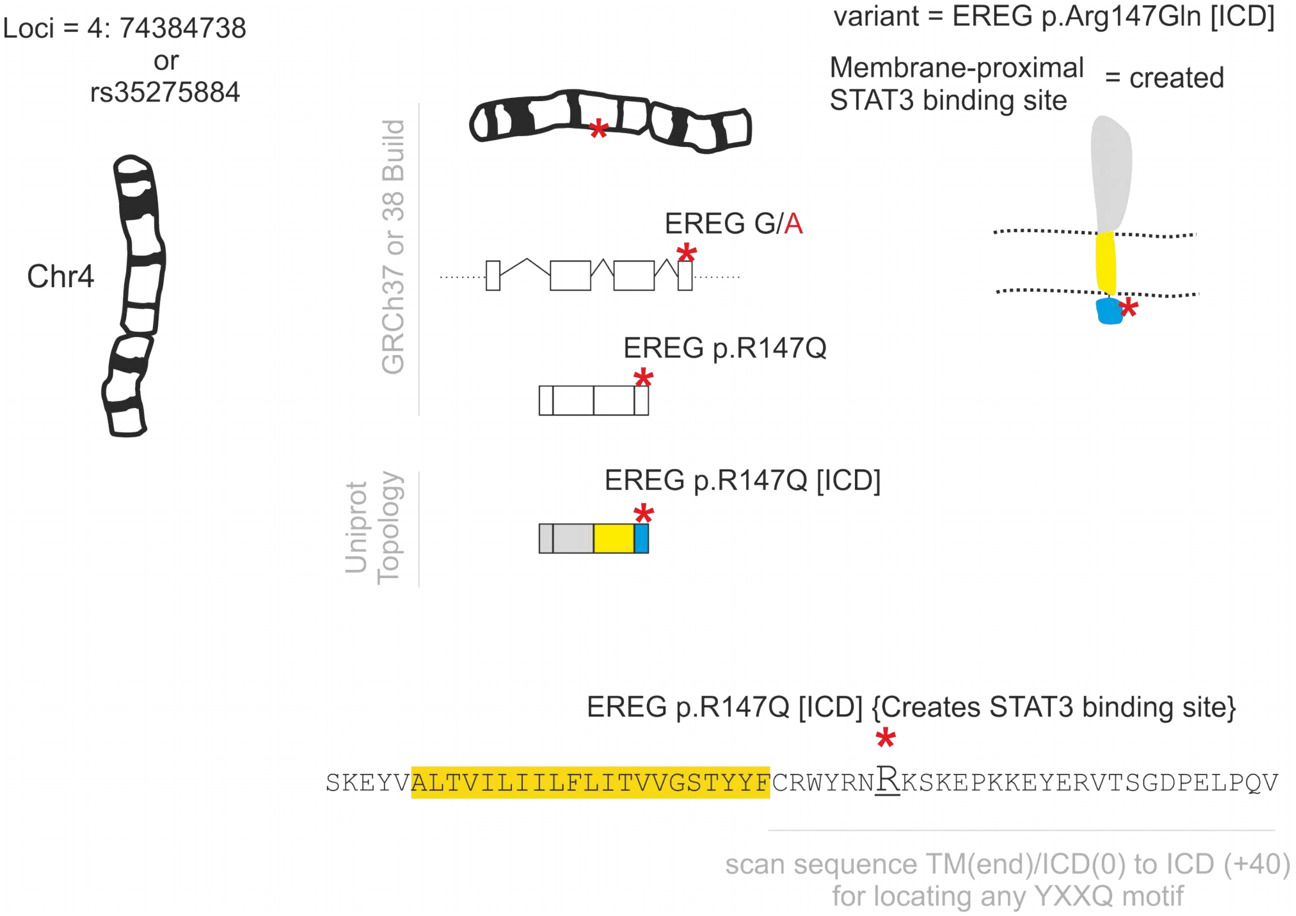

Supplementary Fig. S2 Identification of de novo creation of membrane-proximal tyrosine motifs

Identification of de novo creation of membrane-proximal tyrosine motifs by the single nucleotide variations. For illustration, a variant of EREG is depicted.

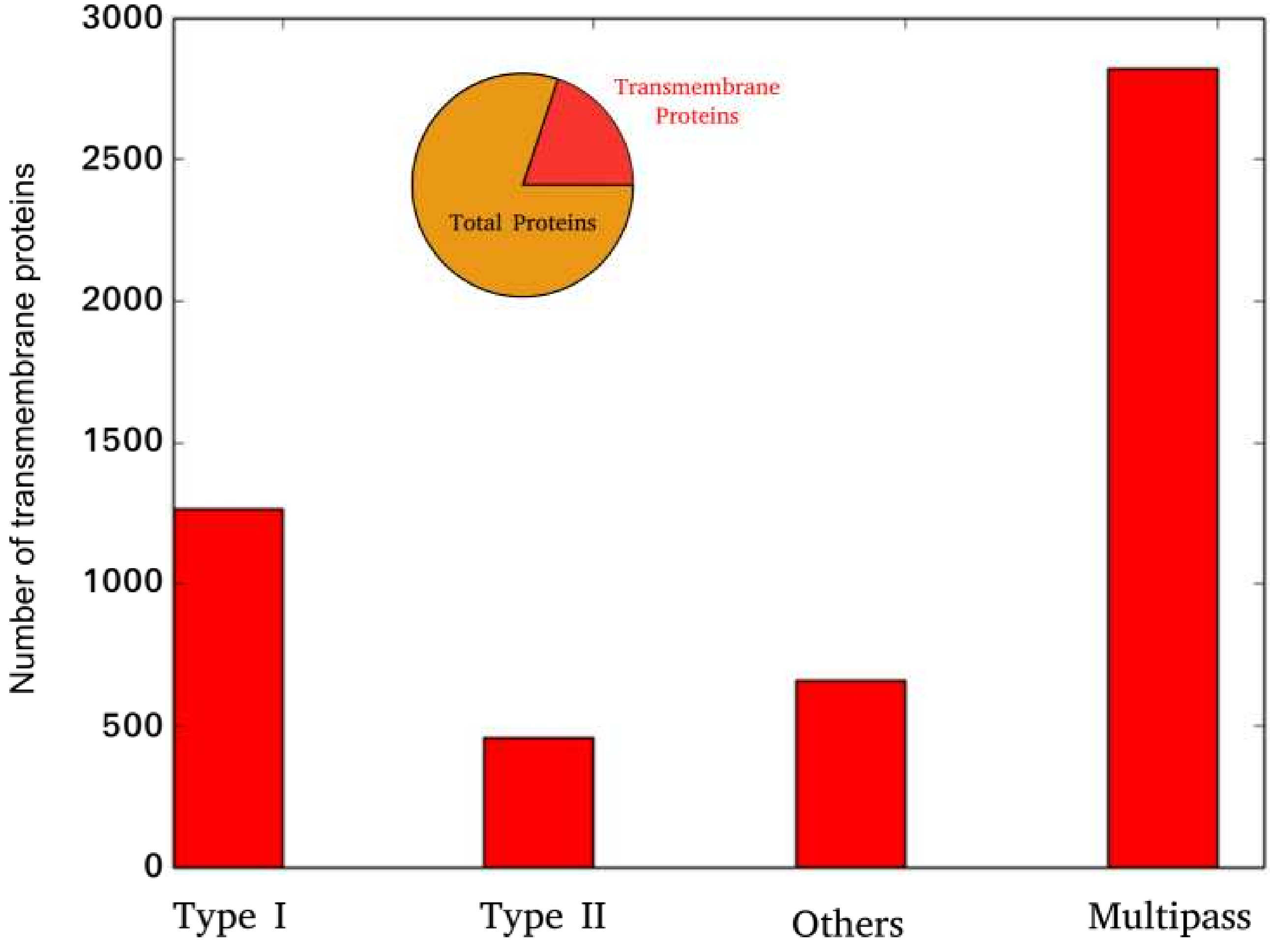

Supplementary Fig. S3 Quantification of human membrane proteins

Numbers of human membrane proteins listed in Uniprot (release February 28, 2020), identified by the presence of at least one transmembrane segment. Pie diagram shows the percentage of transmembrane proteins in the human genome.

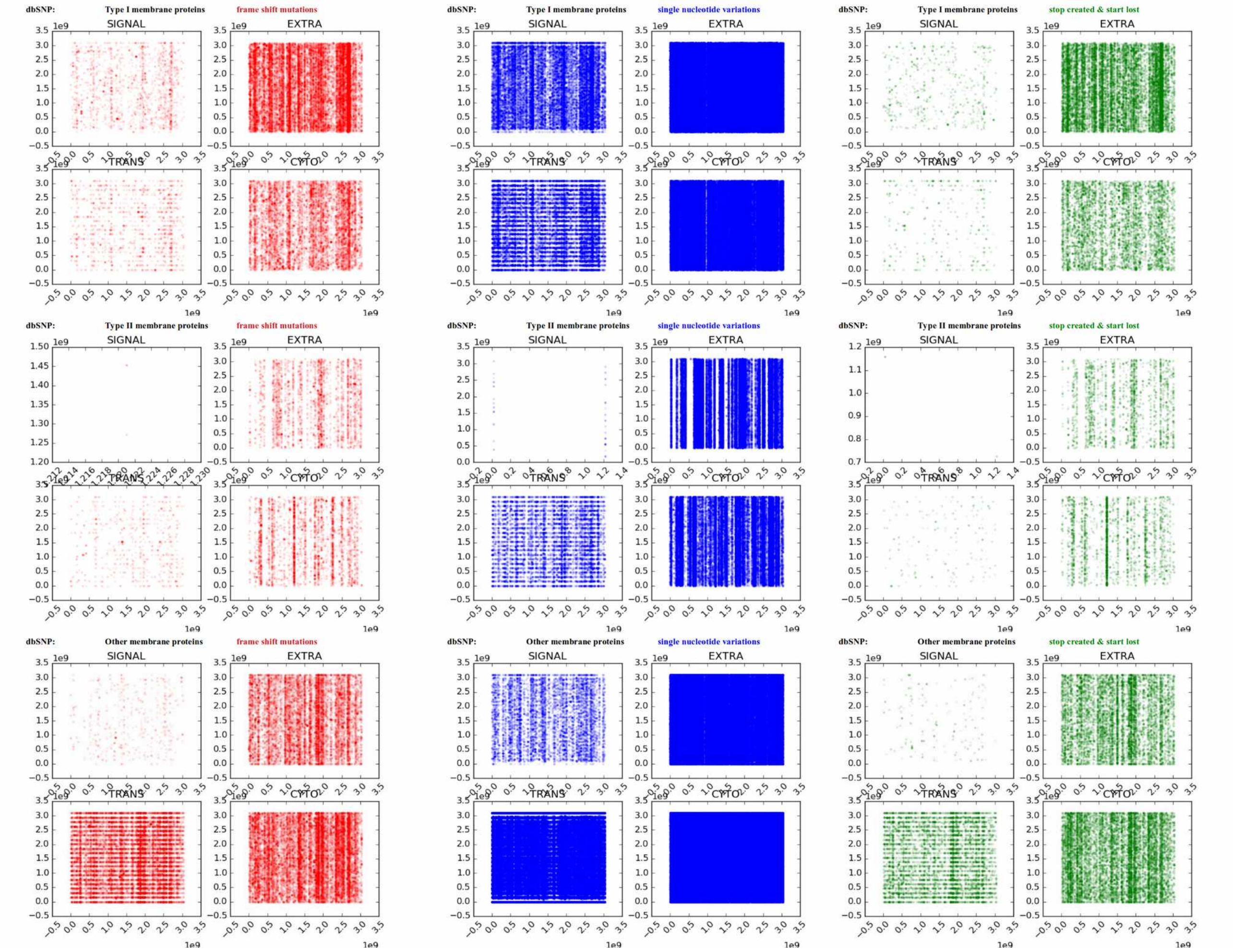

Supplementary Fig. S4 Scatter plots depicting dbSNP data mapped to the domains of membrane proteins

Scatter plots for genetic mutations mapped to the coding regions of human membrane proteins. The chromosome position loci is plotted on the abscissa and normalized domain lengths along the ordinate axes. For each domains namely Signal, Extracellular, Transmembrane and Cytoplasm separate scatter plots are depicted. Each dots represents a genetic variant recorded in vcf files with colour coding namely, frameshift mutations (red), and missense mutations (blue) and stop created mutations (green). Genotyping datasets analyzed here were obtained from dbSNP.

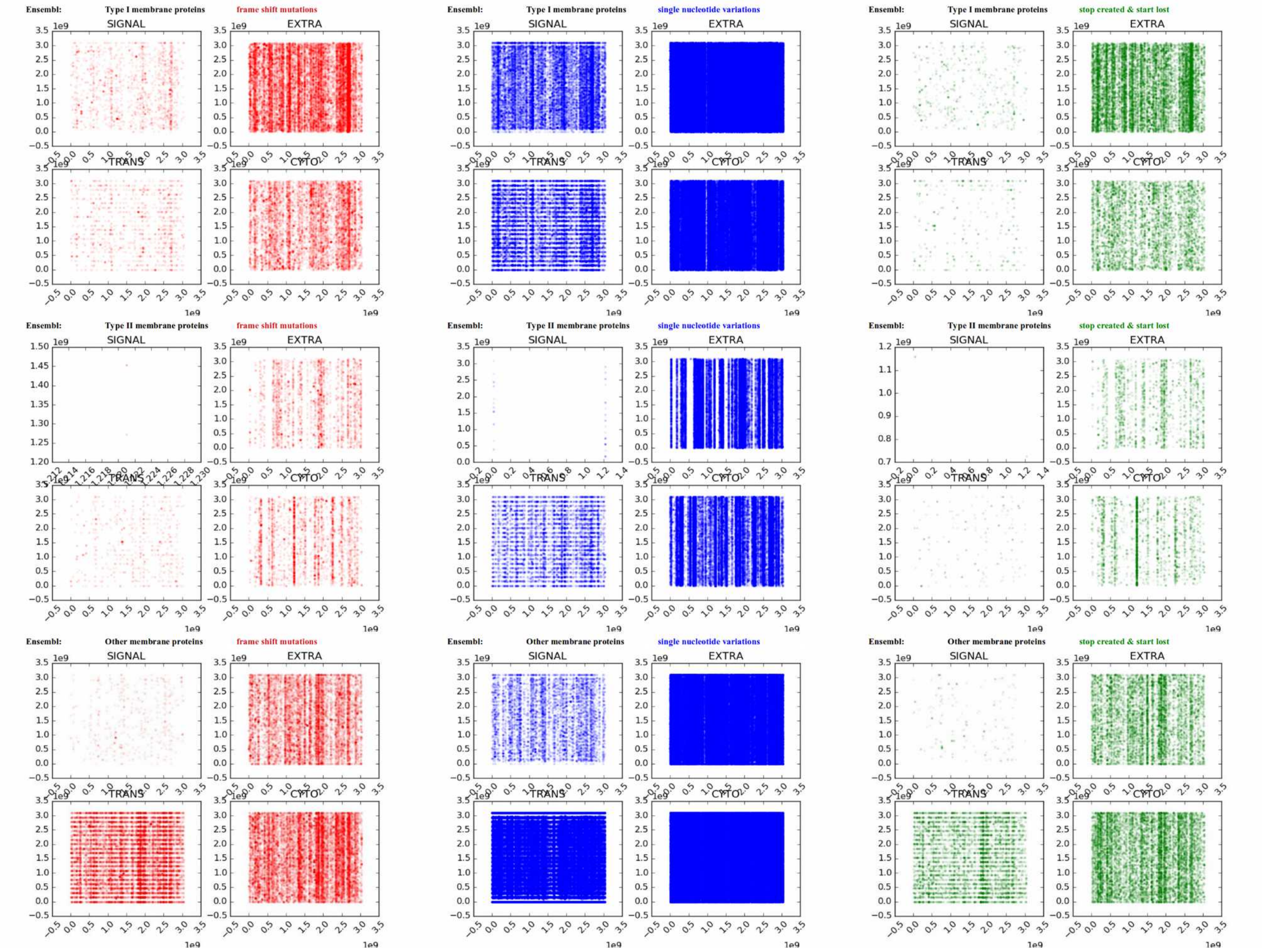

Supplementary Fig. S5 Scatter plots depicting Ensembl data mapped to the domains of membrane proteins

Scatter plots for genetic mutations mapped to the coding regions of human membrane proteins. The chromosome position loci is plotted on the abscissa and normalized domain lengths along the ordinate axes. For each domains namely Signal, Extracellular, Transmembrane and Cytoplasm separate scatter plots are depicted. Each dots represents a genetic variant recorded in vcf files with colour coding namely, frameshift mutations (red), and missense mutations (blue) and stop created mutations (green). Genotyping datasets analyzed here were obtained from Ensembl.

ESP_African American: Type I membrane proteins frame shift mutations

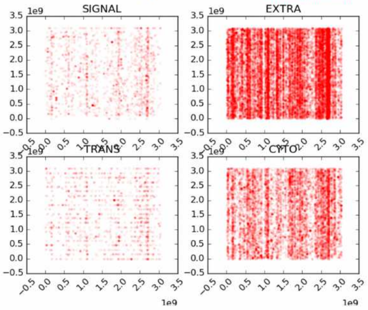

ESP_African American: Type I membrane proteins single nucleotide variations

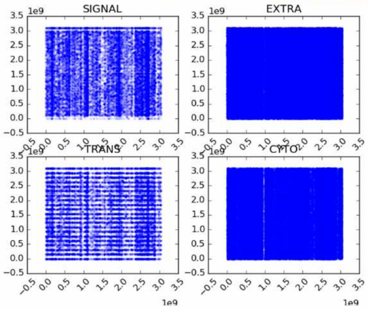

ESP_African American: Type I membrane proteins stop created & start lost

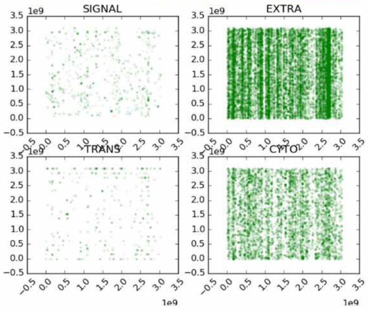

ESP_African American: Type II membrane proteins frame shift mutations

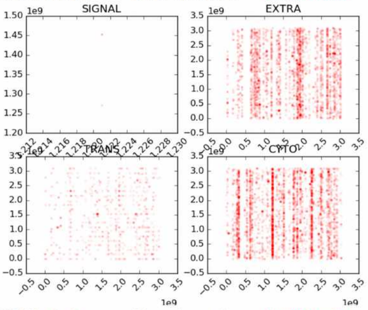

ESP_African American: Type II membrane proteins single nucleotide variations

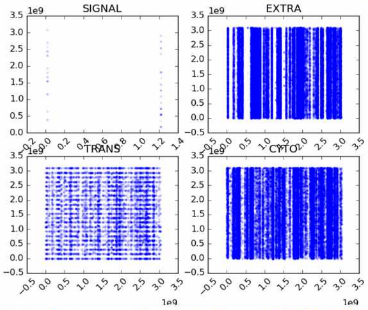

ESP_African American: Type II membrane proteins stop created & start lost

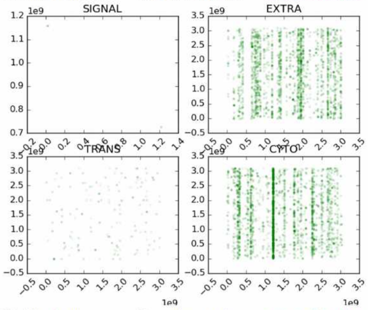

ESP_African American: Other membrane proteins frame shift mutations

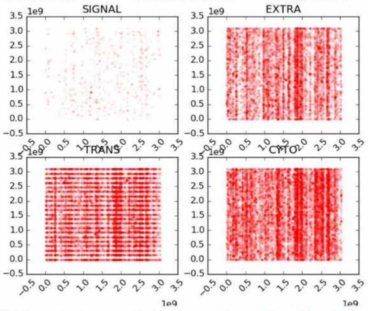

ESP_African American: Other membrane proteins single nucleotide variations

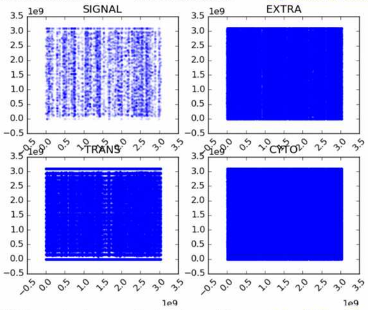

ESP_African American: Other membrane proteins stop created & start lost

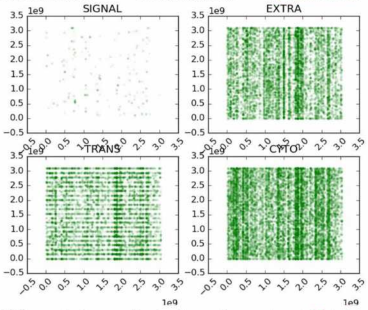

ESP_European American: Type I membrane proteins frame shift mutations

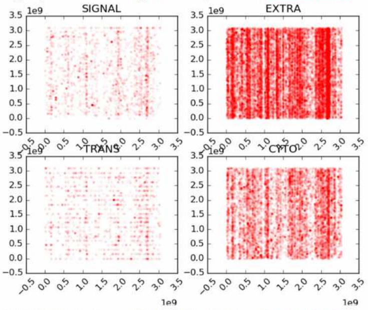

ESP_European American: Type I membrane proteins single nucleotide variations

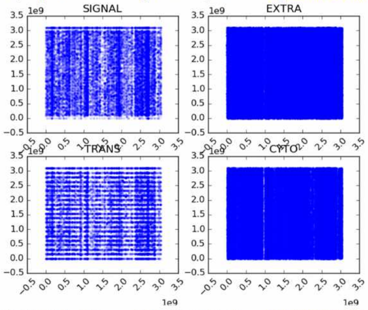

ESP_European American: Type I membrane proteins stop created & start lost

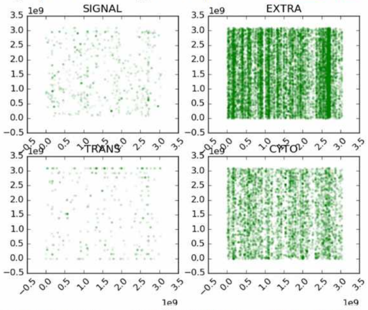

ESP_European American: Type II membrane proteins frame shift mutations

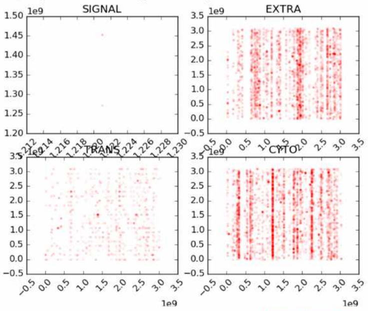

ESP_European American: Type II membrane proteins single nucleotide variations

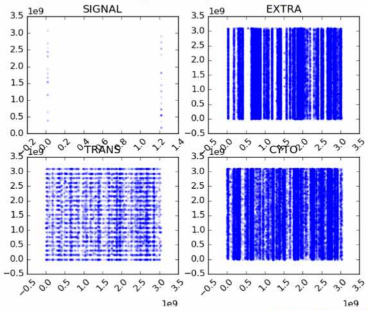

ESP_European American: Type II membrane proteins stop created & start lost

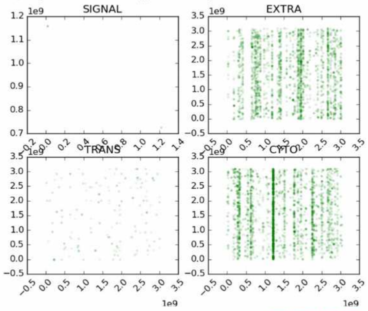

ESP_European American: Other membrane proteins frame shift mutations

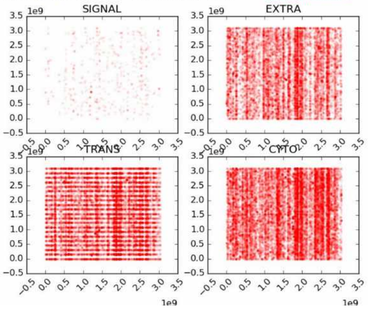

ESP_European American: Other membrane proteins single nucleotide variations

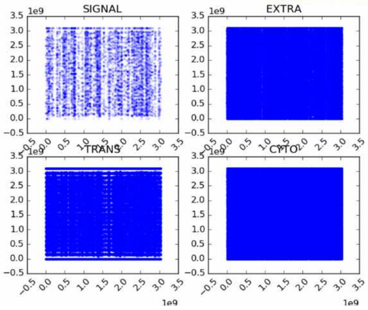

ESP_European American: Other membrane proteins stop created & start lost

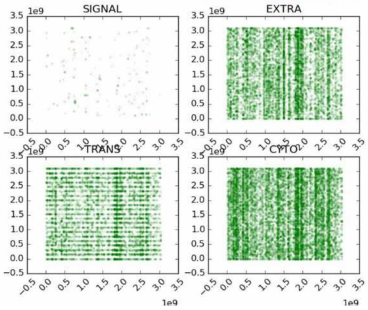

Supplementary Fig. S6 Scatter plots depicting NHLBI Exome Sequencing Project data mapped to the domains of membrane proteins

Scatter plots for genetic mutations mapped to the coding regions of human membrane proteins. The chromosome position loci is plotted on the abscissa and normalized domain lengths along the ordinate axes. For each domains namely Signal, Extracellular, Transmembrane and Cytoplasm separate scatter plots are depicted. Each dots represents a genetic variant recorded in vcf files with colour coding namely, frameshift mutations (red), and missense mutations (blue) and stop created mutations (green). Genotyping datasets analyzed here were obtained from NHLBI Exome Sequencing Project.

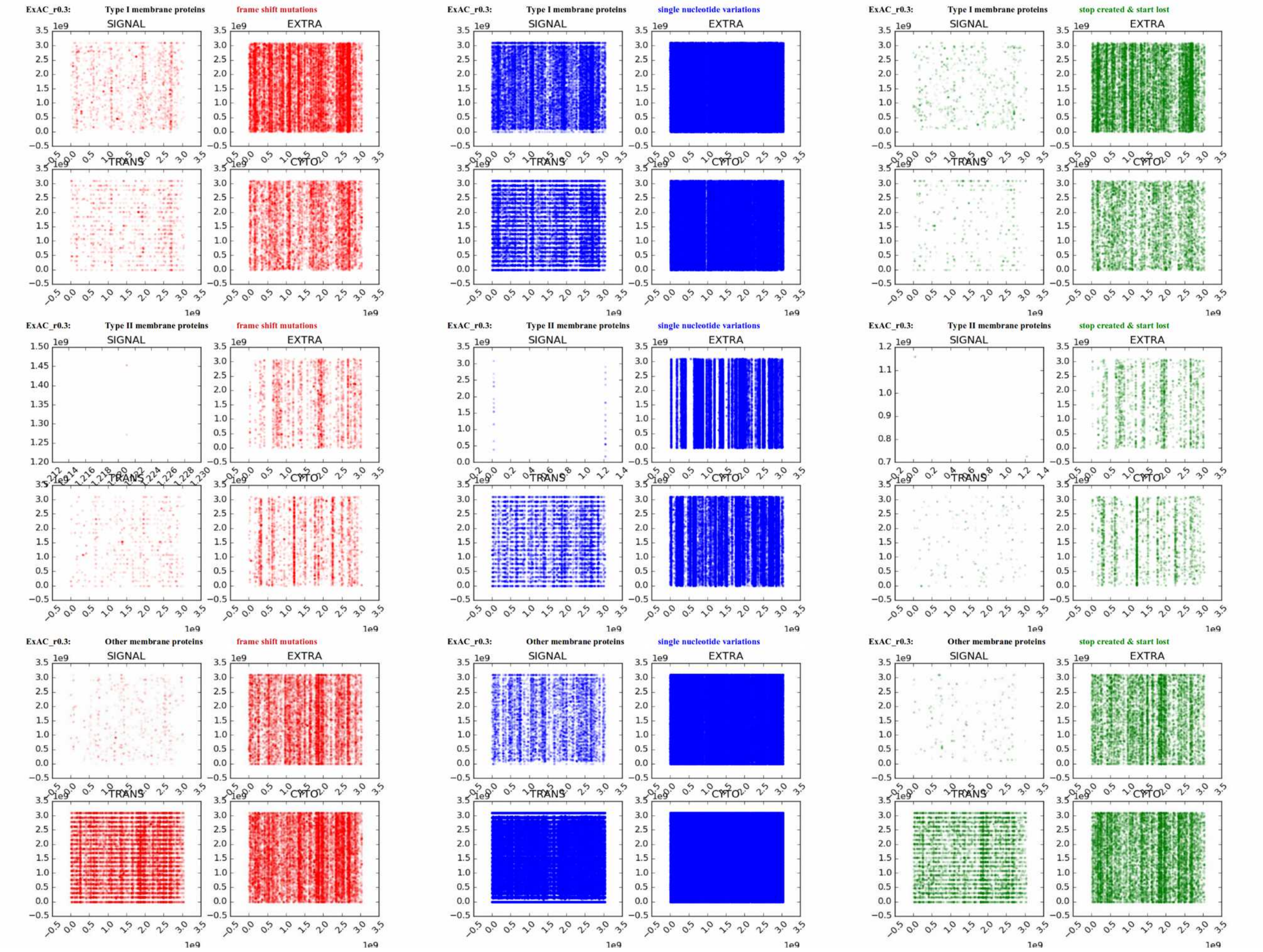

Supplementary Fig. S7 Scatter plots depicting Exome Aggregation Consortium data mapped to the domains of membrane proteins

Scatter plots for genetic mutations mapped to the coding regions of human membrane proteins. The chromosome position loci is plotted on the abscissa and normalized domain lengths along the ordinate axes. For each domains namely Signal, Extracellular, Transmembrane and Cytoplasm separate scatter plots are depicted. Each dots represents a genetic variant recorded in vcf files with colour coding namely, frameshift mutations (red), and missense mutations (blue) and stop created mutations (green). Genotyping datasets analyzed here were obtained from Exome Aggregation Consortium, ExAC r0.3.

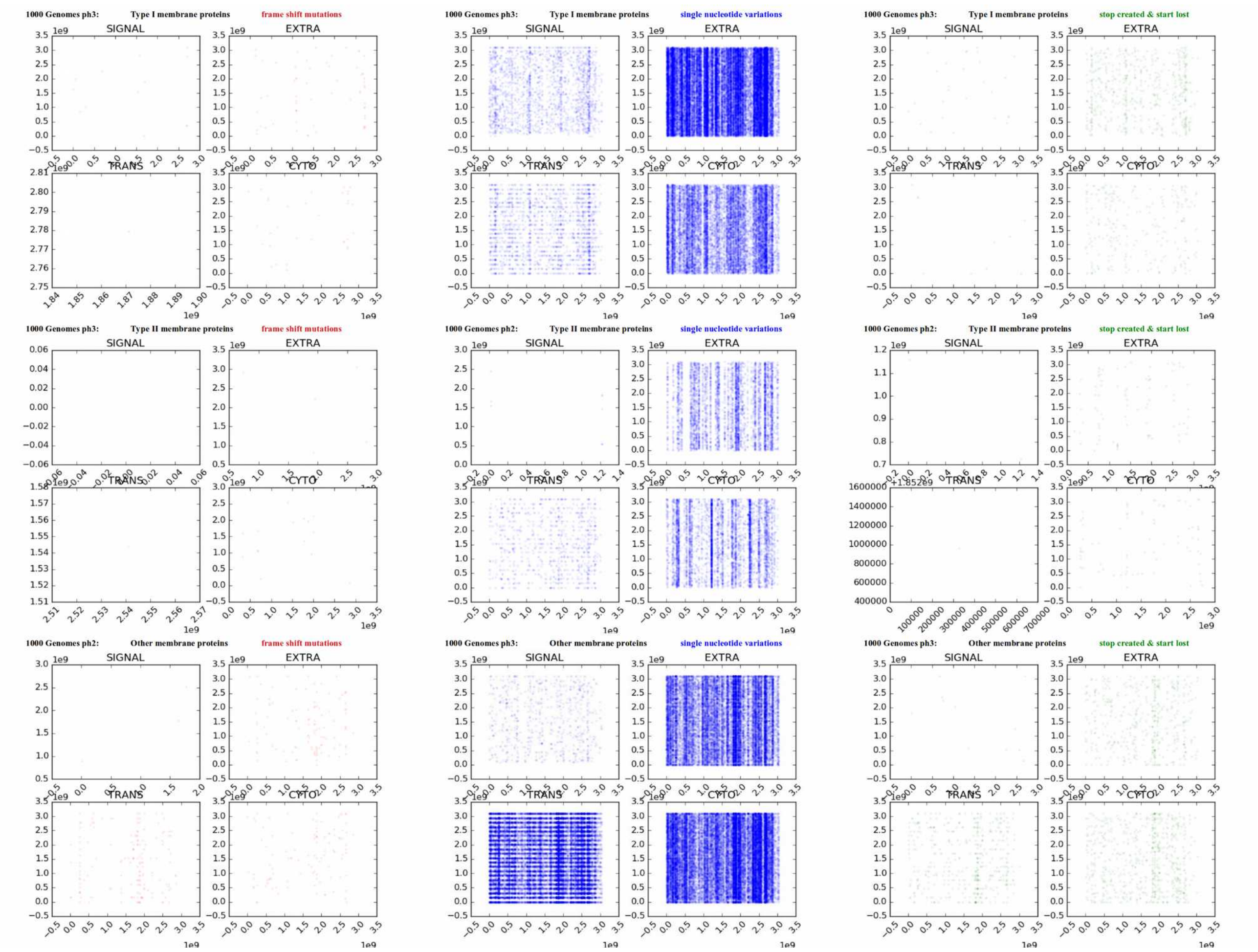

Supplementary Fig. S8 Scatter plots depicting 1000 genome phase 3 data mapped to the domains of membrane proteins

Scatter plots for genetic mutations mapped to the coding regions of human membrane proteins. The chromosome position loci is plotted on the abscissa and normalized domain lengths along the ordinate axes. For each domains namely Signal, Extracellular, Transmembrane and Cytoplasm separate scatter plots are depicted. Each dots represents a genetic variant recorded in vcf files with colour coding namely, frameshift mutations (red), and missense mutations (blue) and stop created mutations (green). Genotyping datasets analyzed here were obtained from 1000 genome phase 3.

107 Figures are accessible:

https://vj-ulaganathan.github.io/Supplemental Figure. 9/Supplementary Figure. 9.html

Supplementary Fig. S9 Scatter plots depicting Hapmap data mapped to the domains of membrane proteins

Scatter plots for genetic mutations mapped to the coding regions of human membrane proteins. The chromosome position loci is plotted on the abscissa and normalized domain lengths along the ordinate axes. For each domains namely Signal, Extracellular, Transmembrane and Cytoplasm separate scatter plots are depicted. Each dots represents a genetic variant recorded in vcf files with colour coding namely, frameshift mutations (red), and missense mutations (blue) and stop created mutations (green). Population groups studied analyzed include (A) ASW - African ancestry in Southwest USA, (B) CEU - Utah residents with Northern and Western European ancestry, (C) CHB - Han Chinese in Beijing, China, (D) CHD - Chinese in metropolitan Denver, Colorado, United States, (E) GIH - Gujarati Indians in Houston, Texas, United States, (F) HCB -Han Chinese, Beijing group, (G) JPT - Japanese in Tokyo, (H) LWK - Luhya in Webuye, Kenya, (I) MEX - Mexican ancestry in Los Angeles, (J) MKK - Maasai in Kinyawa, Kenya, (K) TSI - Toscans in Italy,and (L) YRI - Yoruba in Ibadan, Nigeria. Genotyping datasets analyzed here were obtained from Human HAPMAP.

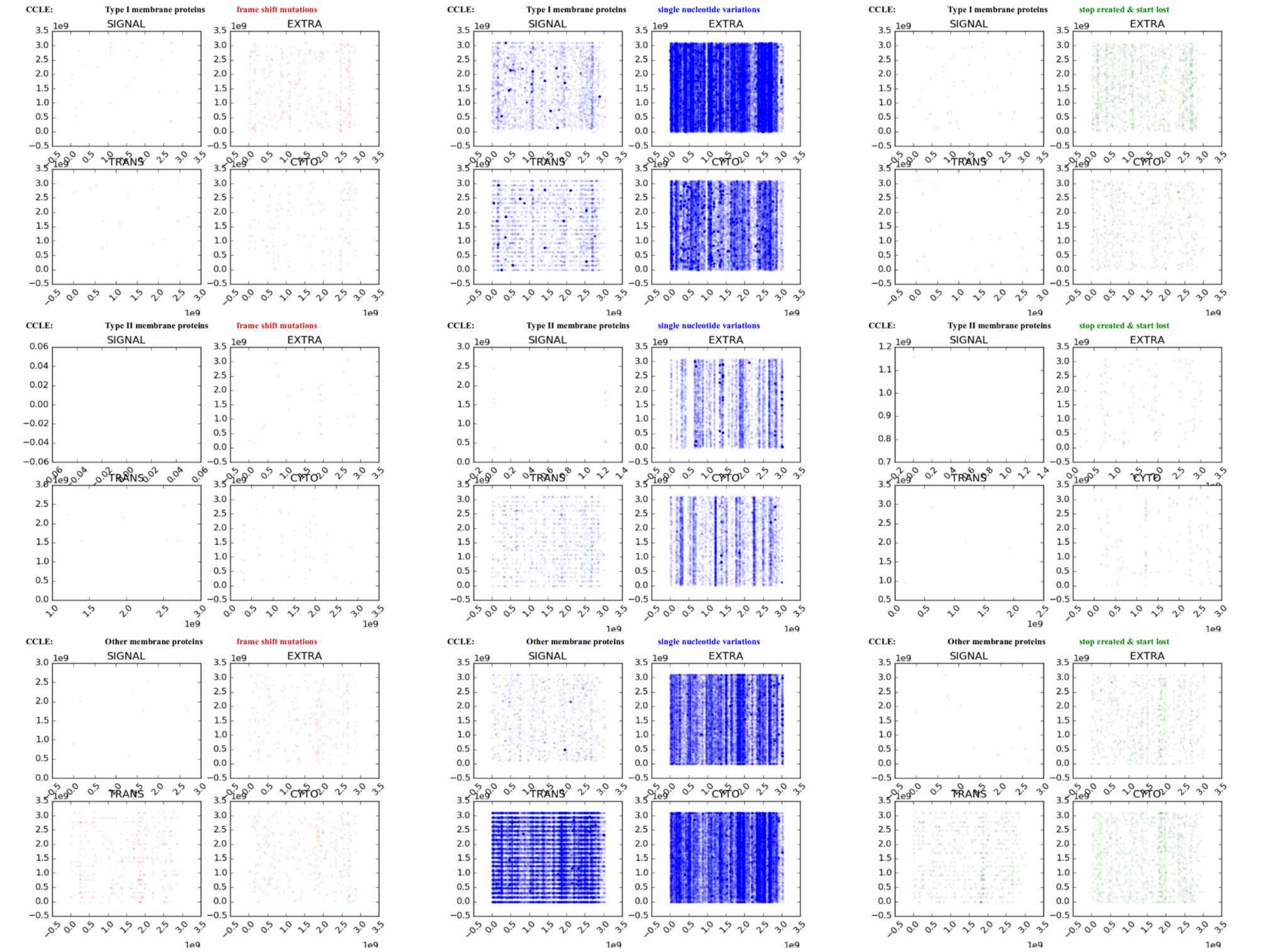

Supplementary Fig. S10 Scatter plots depicting Cancer Cell Line Encyclopedia data mapped to the domains of membrane proteins

Scatter plots for genetic mutations mapped to the coding regions of human membrane proteins. The chromosome position loci is plotted on the abscissa and normalized domain lengths along the ordinate axes. For each domains namely Signal, Extracellular, Transmembrane and Cytoplasm separate scatter plots are depicted. Each dots represents a genetic variant recorded in vcf files with colour coding namely, frameshift mutations (red), and missense mutations (blue) and stop created mutations (green). Genotyping datasets data analyzed here were obtained from Cancer Cell Line Encyclopedia.

26 Figures are accessible:

https://vj-ulaganathan.github.io/Supplemental Figure. 11/Supplementary Figure 11.html

Supplementary Fig. S11 Scatter plots depicting COSMIC data mapped to the domains of membrane proteins

Scatter plots for genetic mutations mapped to the coding regions of human membrane proteins. The chromosome position loci is plotted on the abscissa and normalized domain lengths along the ordinate axes. For each domains namely Signal, Extracellular, Transmembrane and Cytoplasm separate scatter plots are depicted. Each dots represents a genetic variant recorded in vcf files with colour coding namely, frameshift mutations (red), and missense mutations (blue) and stop created mutations (green). Genotyping datasets including Sanger (A) Cell lines, (B) COSMIC and (C) Whole Genome Sequencing data analyzed here were obtained from Sanger COSMIC.

323 Figures are accessible:

https://vj-ulaganathan.github.io/Supplemental Figure. 12/Supplementary Figure. 12.html

Supplementary Fig. S12 Scatter plots depicting the TCGA data mapped to the domains of membrane proteins

Scatter plots for genetic mutations mapped to the coding regions of human membrane proteins. The chromosome position loci is plotted on the abscissa and normalized domain lengths along the ordinate axes. For each domains namely Signal, Extracellular, Transmembrane and Cytoplasm separate scatter plots are depicted. Each dots represents a genetic variant recorded in vcf files with colour coding namely, frameshift mutations (red), and missense mutations (blue) and stop created mutations (green). Genotyping datasets analyzed here were obtained from the TCGA.

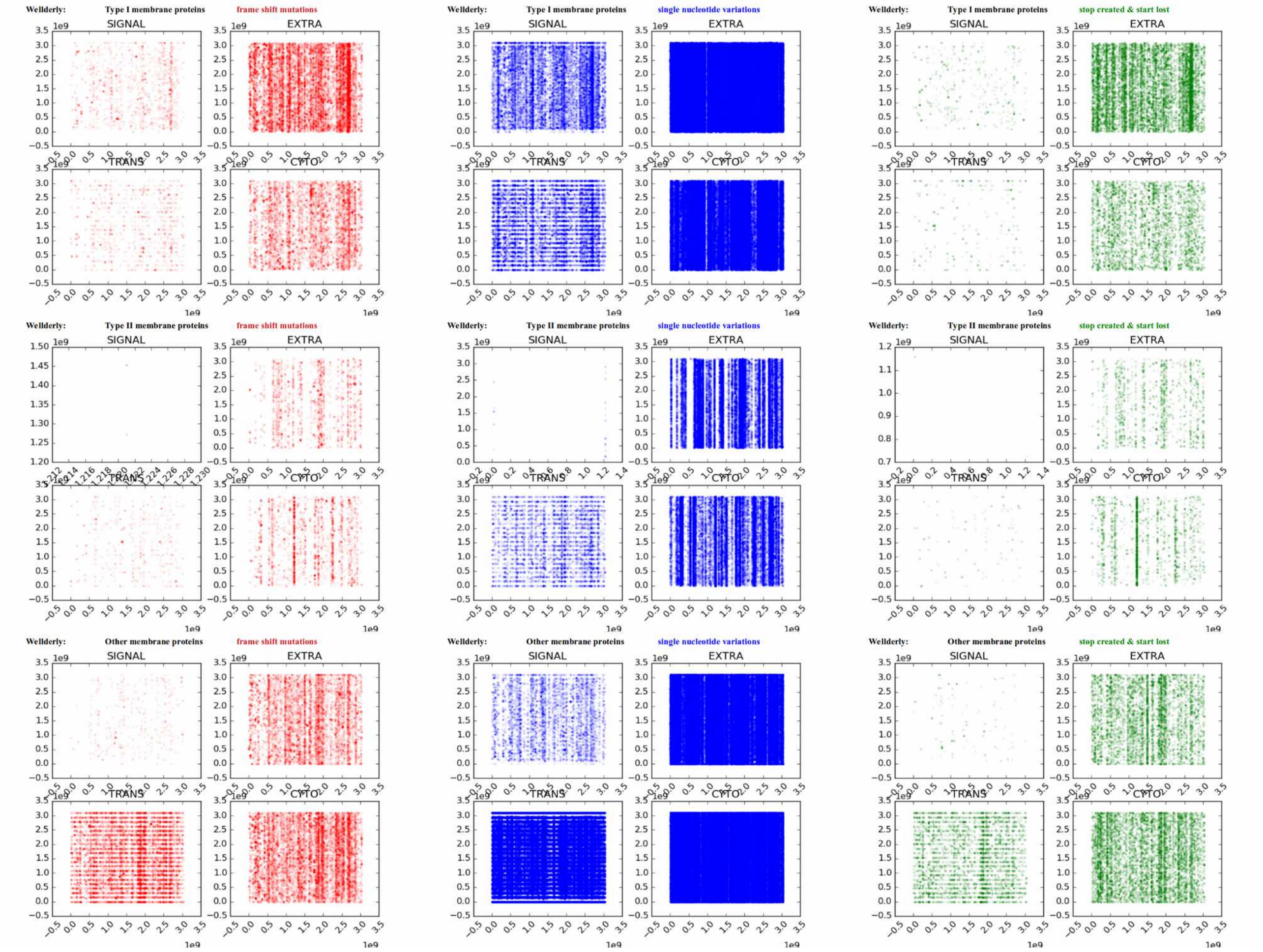

Supplementary Fig. S13 Scatter plots depicting Scripps Wellderly Project data mapped to the domains of membrane proteins

Scatter plots for genetic mutations mapped to the coding regions of human membrane proteins. The chromosome position loci is plotted on the abscissa and normalized domain lengths along the ordinate axes. For each domains namely Signal, Extracellular, Transmembrane and Cytoplasm separate scatter plots are depicted. Each dots represents a genetic variant recorded in vcf files with colour coding namely, frameshift mutations (red), and missense mutations (blue) and stop created mutations (green). Genotyping datasets analyzed here were obtained from the Scripps Wellderly Project.

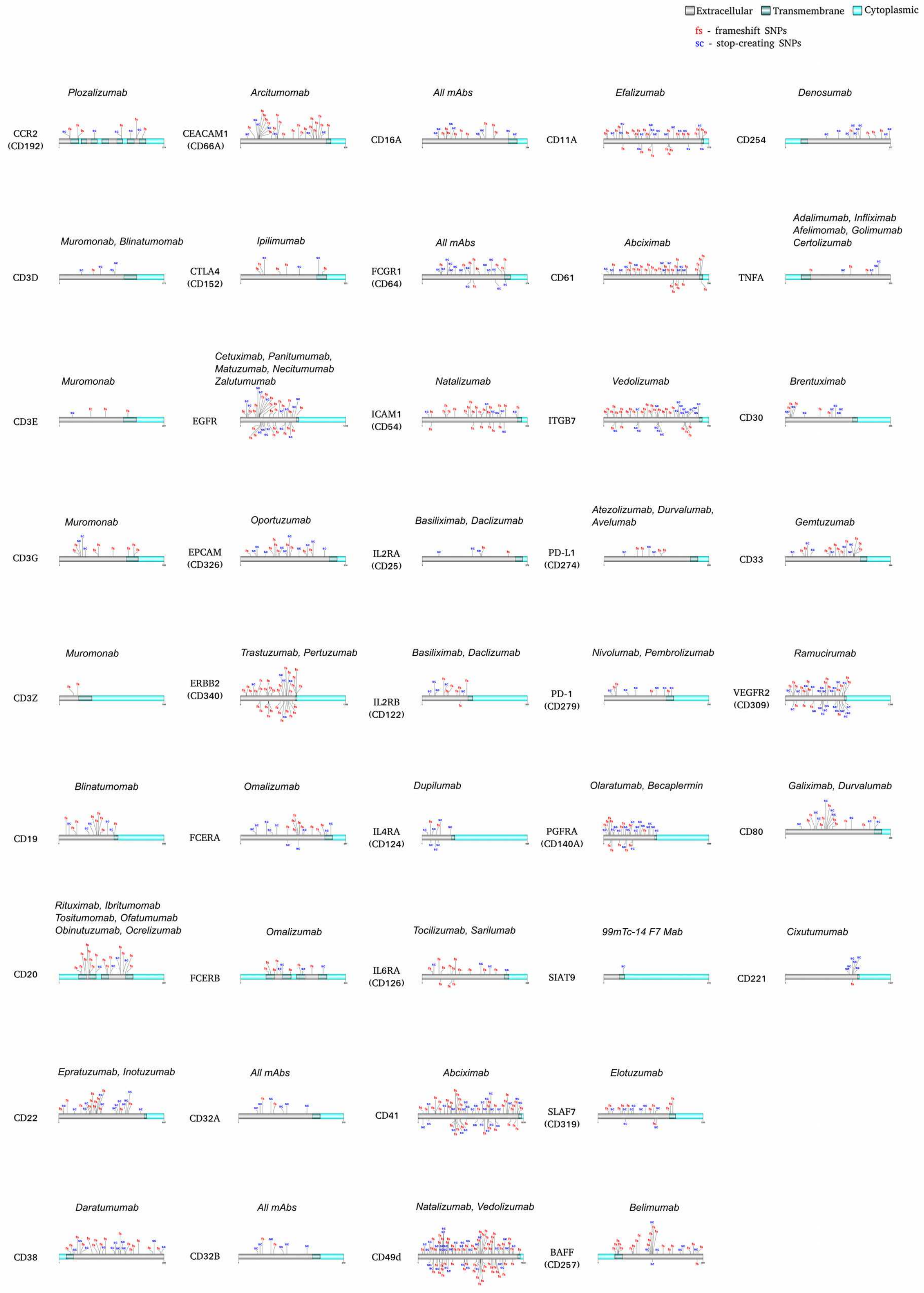

Supplementary Fig. S14 Deleterious mutations altering binding sites of FDA-approved monoclonal antibodies

Schematic illustrations depicting the mapping of frameshift and stop creating genetic variants affecting the extracellular domains of human membrane proteins targeted by FDA-approved monoclonal antibodies. The presence of such germline variants may compromise the therapeutic efficacy and the therapeutic outcomes of clinically approved therapeutic agents.

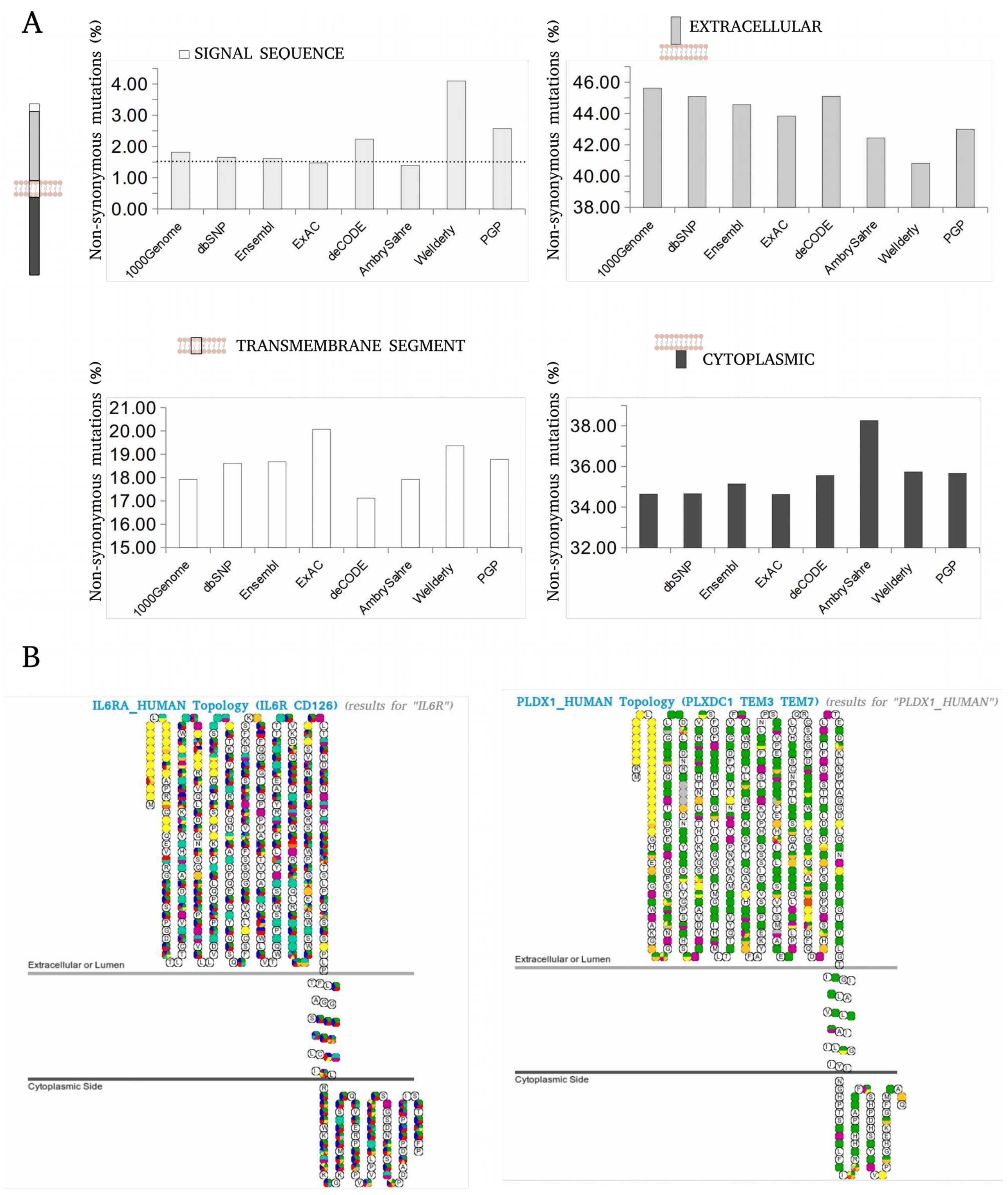

Supplementary Fig. S15 Distribution of mutations on various domains of transmembrane proteins

(A) Prevalence of non-synonymous genetic variations encoding human membrane proteins across signal sequence, extracellular, transmembrane and cytoplasmic segments. Shown are the percentages of mutations for each segments over the total membrane protein variants identified in various studies namely 1000Genome (n=3115), dbSNP, Ensembl, ExAC (n=60,706), deCODE, AmbryShare (n=11400), Wellderly (n=674) and PGP (n=73) where n is number of individual genomes.

(B) Human genetic variation profiles for human IL6RA and PLDX1 proteins. When a variation was identified by multiple studies a multi-coloured circles are depicted where yellow denotes results from Wellderly studies.

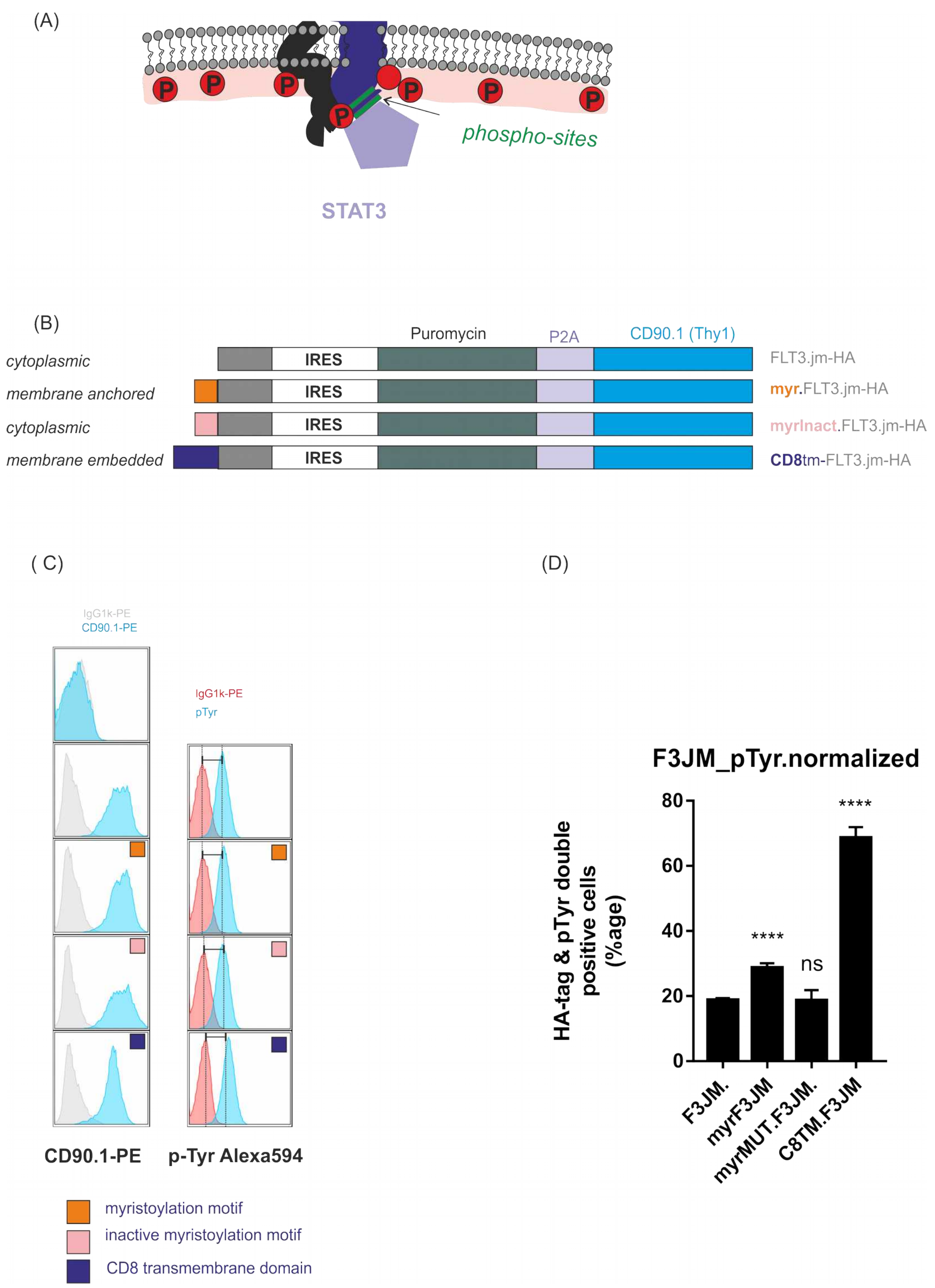

Supplementary Fig. S16 Gain-of-function by STAT3-recruiting YxxQ motifs left intact in the truncated FLT3 p.I638* variant

(A) Illustration of direct recruitment of STAT3 to inner cell membrane by membrane-proximal phosphotyrosine motifs.

(B) Schema depicting polycistronic gene expression constructs for targeted expression of FLT3.jm.

(C) & (D) Assessment of phosphotyrosine levels by intracellular staining for HA-tag and phosphotyrosine gated on CD90.1 expressing HEK293T cells. (C) Overlay histogram relative to isotype staining control and (D) Percentage of HA-tag and phosphotyrosine expressing double positive cells are shown.

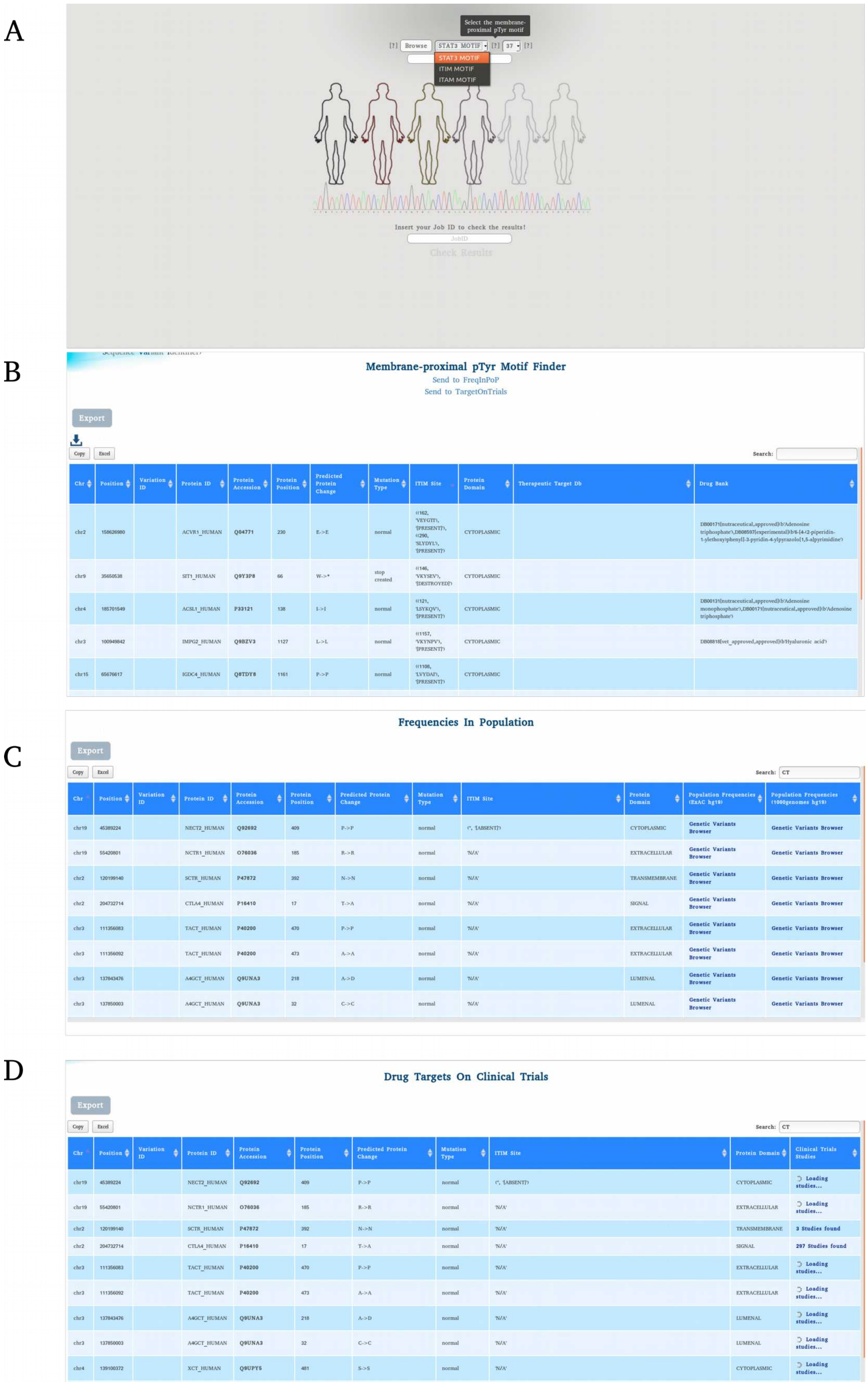

Supplementary Fig. S17 Usage of TraPS-VarI

The TraPS-VarI web application (A) motif selection and upload (B) ‘Motif finder’ results table with links for reanalysis (C) Allele frequency dispenser (D) Clinical trails studies.

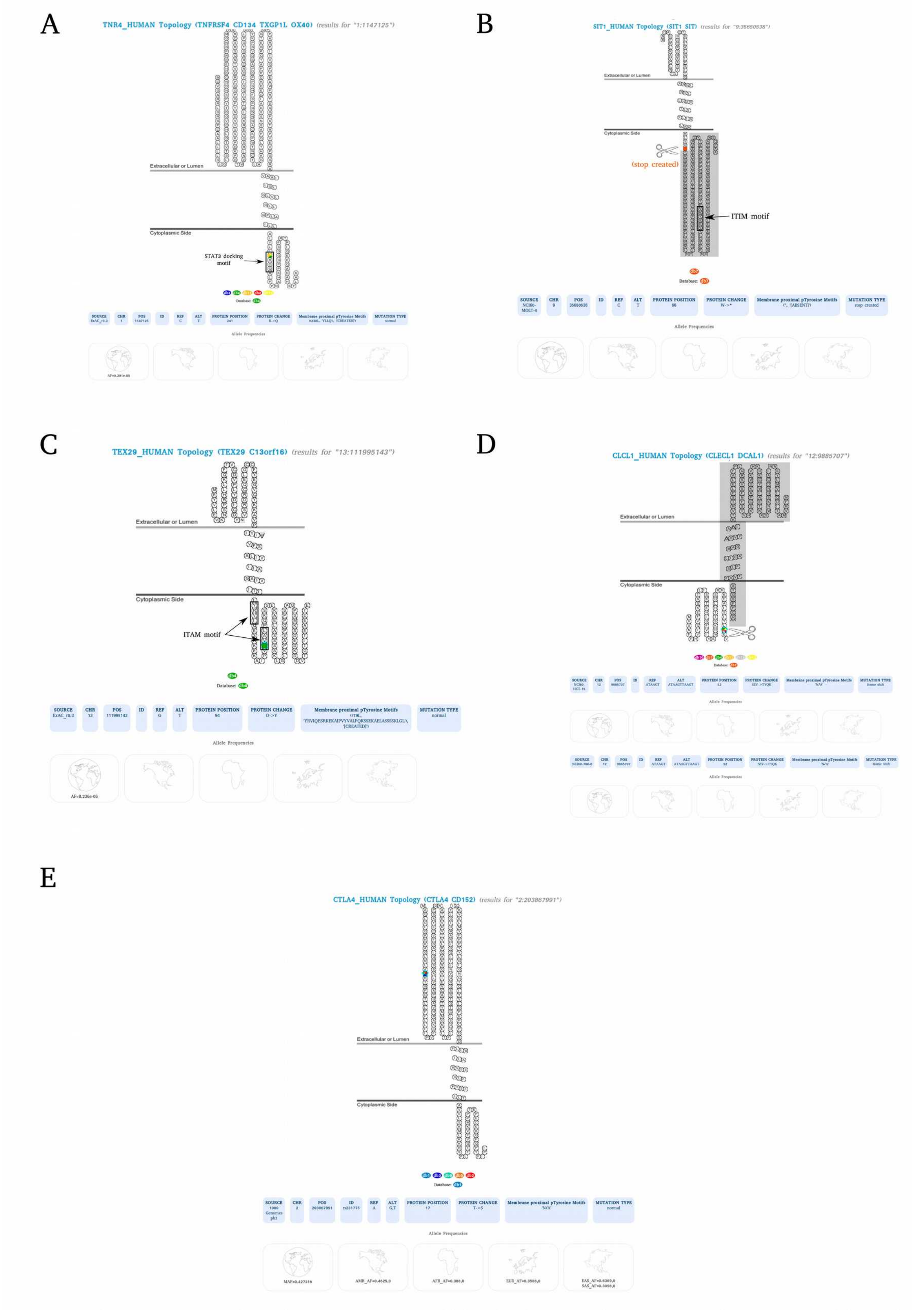

Supplementary Fig. S18 Usage of TraPS-VarI Database Browser

The TraPS-VarI Database Browser facilitates immunologically relevant surface molecule identification. Genetic variant of (A) CD134 with a STAT3 docking site, (B) SIT lacking ITIM motif, (C) TEX29 with a ITAM motif, (D) CLECL1 lacking cell surface expression and (E) CTLA4 with a signal peptide alteration.
